# *In silico* formulation of a next-generation multiepitope vaccine for use as a prophylactic candidate against Crimean-Congo hemorrhagic fever

**DOI:** 10.1186/s12916-023-02750-9

**Published:** 2023-02-01

**Authors:** Rahat Alam, Abdus Samad, Foysal Ahammad, Suza Mohammad Nur, Ahad Amer Alsaiari, Raihan Rahman Imon, Md. Enamul Kabir Talukder, Zulkar Nain, Md. Mashiar Rahman, Farhan Mohammad, Tomasz M. Karpiński

**Affiliations:** 1 Department of Genetic Engineering and Biotechnology, Jashore University of Science and Technology, Jashore, 7408 Bangladesh; 2 Laboratory of Computational Biology, Biological Solution Centre (BioSol Centre), Jashore, 7408 Bangladesh; 3 Division of Biological and Biomedical Sciences (BBS), College of Health and Life Sciences (CHLS), Hamad Bin Khalifa University (HBKU), 34110 Doha, Qatar; 4 Department of Biochemistry, School of Medicine Case, Western Reserve University, Cleveland, OH 44106 USA; 5 College of Applied Medical Science, Clinical Laboratories Science Department, Taif University, Taif, 21944 Saudi Arabia; 6 School of Biomedical Sciences, Baylor College of Medicine, Houston, TX 77030 USA; 7 Chair and Department of Medical Microbiology, Poznań University of Medical Sciences, Rokietnicka 10, 60-806 Poznań, Poland

**Keywords:** CCHF, Multiepitope vaccine, Immunoinformatics, Immune simulation, Molecular dynamics simulation, Molecular docking

## Abstract

**Background:**

Crimean-Congo hemorrhagic fever (CCHF) is a widespread disease transmitted to humans and livestock animals through the bite of infected ticks or close contact with infected persons’ blood, organs, or other bodily fluids. The virus is responsible for severe viral hemorrhagic fever outbreaks, with a case fatality rate of up to 40%. Despite having the highest fatality rate of the virus, a suitable treatment option or vaccination has not been developed yet. Therefore, this study aimed to formulate a multiepitope vaccine against CCHF through computational vaccine design approaches.

**Methods:**

The glycoprotein, nucleoprotein, and RNA-dependent RNA polymerase of CCHF were utilized to determine ﻿immunodominant﻿ T- and B-cell epitopes. Subsequently, an integrative computational vaccinology approach was used to formulate a multi-epitopes vaccine candidate against the virus.

**Results:**

After rigorous assessment, a multiepitope vaccine was constructed, which was antigenic, immunogenic, and non-allergenic with desired physicochemical properties. Molecular dynamics (MD) simulations of the vaccine-receptor complex show strong stability of the vaccine candidates to the targeted immune receptor. Additionally, the immune simulation of the vaccine candidates found that the vaccine could trigger real-life-like immune responses upon administration to humans.

**Conclusions:**

Finally, we concluded that the formulated multiepitope vaccine candidates would provide excellent prophylactic properties against CCHF.

**Supplementary Information:**

The online version contains supplementary material available at 10.1186/s12916-023-02750-9.

## Background

Crimean-Congo hemorrhagic fever (CCHF) is caused by a virus transmitted to humans by the bites of infected ticks *Hyalomma *spp. [1, 2]. CCHF causes a mild febrile illness that may progress to severe and often fatal hemorrhagic shock responsible for multiple organ failures in humans and animals [1, 3]. The virus was first detected in the Crimean region of the Soviet Union in 1944 and later in Congo in 1969 and hence was given the name CCHF [4]. Until now, the virus has been causing sporadic cases or outbreaks in Asia, Africa, the Middle East, and Eastern Europe [5]. CCHF belongs to the genus *Nairovirus* in the *Bunyaviridae* family [2]. Structurally, the virus is spheroid-shaped (~ 80–100 nm in diameter) and contains G_C_ and G_N_ spike glycoproteins in the lipid envelope. The virions carry a single-stranded genomic RNA with three genomic segments including small (S), medium (M), and large (L) segments. These genomic segments are further encapsulated by the nucleoprotein (NP) and an RNA-dependent RNA polymerase (RdRp), where the S, M, and L genomic segments encode for G_n_/G_c_ nucleoprotein [4, 6].

The main target of CCHF infections is hepatic endothelial cells, hepatocytes, and Kupffer cells. Clinically, the CCHF illness spectrum is very broad. The main contributing factors leading to fatality are critical anemia, cerebral hemorrhage, extreme dehydration, lung edema, myocardial infarction, and multi-organ failure, including liver, cerebral, and kidney dysfunction and pulmonary and cardiac shortfall [7]. The CCHF patients also have higher blood ALT, AST, CK, LDH, PT, and aPTT indications [8]. A recent report has also suggested that increased levels of proinflammatory cytokines, like IL-1, IL-6, and TNF-α, play a major role in the mortality of CCHF patients [3]. To date, no specific anti-viral therapy or approved vaccine has been designed to treat or prevent infection that occurred through the virus. However, corticosteroid medications (dexamethasone and methylprednisolone), immunotherapy, neutralizing antibodies [9], and convalescent serum [10] are being considered as medication for CCHF. Among therapeutics, vaccines are proven to be most effective in combating infectious diseases, and rationally designed peptide vaccines have shown promising results in inducing a specific immune response. The immunodominant T or B cell epitope is the key basis for epitope-based peptide vaccine designing that can elicit a protective and specific immune response against rapidly evolving infectious diseases [11, 12]. Multiepitope-based vaccine design has been becoming an attractive strategy and has been employed against various viral diseases like influenza and dengue virus [13, 14]. Compared to multiepitope-based vaccines, the traditional vaccine design approaches target the whole proteomes of organisms and lead to undesirable antigenic responses, which may cause allergic reactions. Instead, short and specific immunogenic peptide-based vaccines may rule out this complication as they can evoke robust and non-allergic immune responses [15]. Recent advances in computational biology tools have proven effective in designing effective vaccines [16]. Moreover, multiepitope-based vaccine design is advantageous over traditional and single epitope vaccines as it comprises multifaceted MHC epitopes, inducing a simultaneous humoral and cytotoxic immune response, and multiple epitopes can cover a wide range of targeted infected cells, etc. [17].

The immune response induced by the multiepitope-based vaccine may be composed of cytotoxic T lymphocytes (CTL), B cells, and T helper (Th)-cell epitopes. Precursor CD8^+^ CTL cell (pCTL CD8 +) perceived peptide antigen (Ag) through T-cell receptor (TCR) that is presented MHC-I expressed by virus-infected cells [17]. On the other hand, antigen-presenting cells (APC) process the multiepitope peptides and present the Ag through MHC-II to CD4 + T cell (Th_0_). The Th_0_ further propagated into Th_1_, Th_2_, and effector CD4 + CTL cells, where Th_1_ secretes cytokines (IL-2/12, IFN-ƴ) which help pCTL CD8 + cells to become effector CTL cells. Additionally, B cells process multiepitope peptides and present Ag to Th2 by B cell receptor (BCR), which further activates B cells by CD40L and cytokines. B cells uptake Ag and present to Th2 through MHC-II. Activated Th2 cell secretes cytokines (IL-4/5/6, TGF-β) to assist B cells to become differentiated into plasma cells to elicit ADCC and CDC actions [16, 17]. Furthermore, surviving CCHF patients showed long-lived CD8 + T cell response after infections which additionally confirmed with IFN-γ productions by T cell epitope [18]. Also, CCHFV nucleoproteins has intrinsic endonuclease activity that produces 5′ppp uncapped RNA, which can cause CCHFV infection and can evoke RIG-I-mediated type I IFN response [19], as well as TLR3/7/8/9, which has a significant role in CCHF infections [20].

The enveloped viruses including CCHFV contain surface glycoproteins G_n_ and G_c_ which are 140-KDa and 85-KDa proteins, respectively [21]. Furthermore, it has been shown that most of these viruses have N glycosylated glycoproteins. In CCHFV, although both G_n_ and G_c_ glycoproteins are N glycosylated, however, only N glycosylation of G_n_ proteins is important for glycoprotein correct folding, trafficking, and localizations [20]. Also, G_n_ is considered very important because it plays a major role in the packaging of the CCHFV genome and virion assembly by its cytoplasmic tail content zinc finger domain that binds with ribonucleoproteins [22, 23]. In a recent study on mice, it has been shown that G_n_ can elicit a potent immunogenic response by producing increased levels of IFNγ and IL-10 [24]. Hence, multiepitope-based vaccines against G_n_ Ag could be a new therapeutic strategy against CCHFV. In this study, we have applied various in silico approaches to formulate a multiepitope vaccine against CCHF. Biological activity was assessed using bioinformatic tools, and using glycoproteins and RdRp, we identified immunodominant T- and B-cell epitopes and designed multiepitope vaccines, which are antigenic, immunogenic, and non-allergenic with suitable physicochemical properties. Furthermore, we show through molecular dynamics (MD) simulations that the vaccine-receptor complex has stability of the vaccine candidates to the desired immune receptor.

## Methods

### Target protein sequence and antigenicity prediction

Three basic structural and functional protein segments—M, S, and L of CCHFV—were retrieved from the ViPR database (https://www.viprbrc.org/). These datasets were further consolidated with data as well as analysis software for various viral families, including human pathogenic viruses. In addition, it also includes protein sequences, annotation of genes and proteins, the structure of 3D proteins, locations for immune epitopes, metadata, etc. [25]. The protein sequences were retrieved in a FASTA format. Since in vaccine development, antigenicity is a crucial step for eliciting an immune response, we first performed antigenicity prediction. For antigenicity prediction, the Vaxijen v2.0 Server (http://www.ddg-pharmfac.net/vaxijen/VaxiJen/VaxiJen.html) has been used. Epitopes with a threshold antigenicity score > 0.4 were considered antigenic [26].

### Screening and validation of cytotoxic T lymphocyte (CTL) epitopes

An anti-viral vaccine is considered ideal if it develops the functional cytotoxic T lymphocytes (CTLs), which are the very critical mediator of adaptive anti-viral immunity [27–29] and that can combat against a vast range of pathogens [30]. Hence, we used NetCTL v1.2 Server (http://www.cbs.dtu.dk/services/NetCTL/), which is an integrated server to predict epitopes for a given protein sequence based on affinity for MHC-I, the transportation efficiency of TAP, and the cleavage of the proteasome [31]. The threshold value for epitope identification was 0.75, and it was sorted out by the combined score. Additionally, the predicted epitopes were further evaluated for immunogenicity prediction through (http://tools.iedb.org/immunogenicity/) [32]. Then, we used ToxinPred Server (http://crdd.osdd.net/raghava/toxinpred/) [33] and Vaxijen v2.0 Server: (http://www.ddg-pharmfac.net/vaxijen/VaxiJen/VaxiJen.html) for evaluating the toxicity and antigenicity, respectively. Furthermore, to check out the status of the allergenicity of predicted epitopes, AllergenFP (https://ddg-pharmfac.net/AllergenFP/) [34] and AllerTOP v. 2.0 (https://www.ddg-pharmfac.net/AllerTOP/) [35] were used. Finally, the binding of the MHC-I allele for CTL epitope was predicted through IEDB MHC-I Allele (http://tools.iedb.org/mhci/).

### Screening and validation of helper T lymphocyte (HTL) epitopes

The HTLs are a critical player in adaptive immune response development and are known to exhibit multifaceted functioning like regulating T and B cells, recognizing foreign molecules through MHC-II on APC, assisting in T cell-mediated immunity, etc. [36]. Additionally, designing the T helper cell epitope is also crucial for effective vaccine development as vaccine Ag becomes processed to be presented through MHC-II [16, 37]. For screening the HTL epitopes and MHC-II binding allele, the IEDB MHC-II Binding (http://tools.iedb.org/mhcii/) web server was used. The percentile rank of 0.5 was applied for counting alleles for the consensus 2.22 prediction method and the immunogenic epitope of 15-mer was selected. The found epitopes were further investigated for the in silico assessment of IL-4, IL-10, and IFNγ, through IL4Pred (https://webs.iiitd.edu.in/raghava/il4pred/), IL-10Pred (http://crdd.osdd.net/raghava/IL-10pred/), and IFNepitope (http://crdd.osdd.net/raghava/ifnepitope/predict.php). Additionally, antigenicity and toxicity were also checked by Vaxijen v2.0 and ToxinPred server.

### Evaluation of population coverage study

The human leukocyte antigen (HLA) alleles are diversified among the human population [38–40]. Population coverage is an important aspect to gauge the efficiency of the selected epitopes. We analyzed the distribution of CTL and HTL epitopes across various populations for assessing the population coverage through IEDB tools (http://epitope.liai.org:8080/tools/population). The tools were highly customized to predict epitope-based vaccines or diagnostics designing that can be maximized the study coverage with minimized complexities and variability found in various ethnic communities [41].

### Mapping of multiepitope vaccines

The multiepitope vaccine was constructed by joining epitopes of HTL and CTL via linkers. Further adjuvants were added into the vaccine construct [42–44] as an adjuvant has been considered an essential part of the vaccine and vigorous immune response augmenter against disease [45–47]. Previously, it has been shown that TLR-3/8 had a significant clinical presentation and susceptibility in CCHF viral disease [48, 49]. Most of the viruses utilize the TLR4 for recognition and entry into the host [50]; therefore, we have used all of the receptors for vaccine construction. For the TLR-3/8 receptor target (PDB ID: 2A0Z, 3W3G), the β-defensin adjuvants were used at the N terminal and accompanied by EAAAK linker to its CTL epitopes [51]. In contrast, for the TLR-4 receptor target (PDB ID: 4G8A), the 50S ribosome adjuvant was used at the N terminal and accompanied by EAAAK linker to its CTL epitopes [52]. In both cases, the CTL and HTL linkers were added with AAY and GPGPG linkers [52, 53].

### Evaluation of physicochemical properties

For the vaccine, antigen-adjuvant interaction is very significant in terms of vaccine safety, and to study different types of physiochemical effects and the intensity of immune response, it is essential to characterize the vaccine antigen-adjuvant physiochemical attributes [54, 55]. The ProtParam web server (https://web.expasy.org/protparam/) [56] was used to calculate physicochemical properties. The antigenicity, immunogenicity, and allergenicity were further evaluated. The solubility also has been checked with the SoLPro web server (http://scratch.proteomics.ics.uci.edu/) [57], which has the capacity of multiple runs of tenfold cross-validation and provides 74% accuracy. The solubility was further validated by Protein-Sol [58] web tools (https://protein-sol.manchester.ac.uk/).

### 3D vaccine modeling, refinement, and evaluation

The RaptorX (http://raptorx.uchicago.edu/) web server was used to predict the tertiary structure of the candidate vaccine construct. The RaptorX is a protein modeling server, which can perform nonlinear alignment scoring functions, assessment of alignment quality, multiple-template threading, probabilistic sampling of alignments, function annotation of structure models, and Domain parsing, etc. [59]. The predicted structure was visualized by Maestro v11.3. The crude structure obtained from RaptorX was further evaluated by refining the structure using GalaxyRefine (http://galaxy.seoklab.org/) web tools, based on the CASP9 approach. The server uses several steps for refining the crude protein model like multiple template selection, using PROMALS3D, Conformational Space Annealing (CSA) optimization of restraint energy, and Unreelable Local Regions (ULR) energy, etc. [60]. Additionally, the vaccine tertiary structure was further validated using ProSA-web (https://prosa.services.came.sbg.ac.at/prosa.php) tools [61]. This server can generate a quality model even from low-resolution input or model with only available C^α^ trace as well as it can provide *z*-score and plotted energies of residues [61]. The *z*-score was analyzed with ProSA-web tools [61]. Hereafter, for assessing the nonbonded interactions, we used the SAVES (https://saves.mbi.ucla.edu/) server [62] followed by creating the Ramachandran plot [63, 64]. The Ramachandran plot manifests the allowed and disallowed region for amino acid moieties and validates protein structure based on φ (phi) and ψ (psi) angles of amino acid [63, 65]. The Ramachandran score was analyzed with SAVES web tools. The initial and refined vaccine’s structure was visualized by Maestro v-11.3.

### Binding affinity of vaccine-receptor complexes

The binding interactions between proteins and desired ligands can be revealed by molecular docking studies [66]. Toll-like receptors 3/8 (TLR-3/8) play a crucial role in CCHF viral infection [48, 67], and TLR4 has critical functioning for initiation of viral pathogenesis to induce inflammatory response [68]. Hence, we chose these TLRs as our receptors and refined the vaccine model as a ligand. The molecular docking study was performed using ClusPro v2.0 web tools (https://cluspro.bu.edu/login.php) [69]. Recently, ClusPro has been reported to be more trustworthy than other docking methods for unbound protein structures [70], most likely due to final selection based on cluster size rather than scoring function value [71, 72]. Before the docking, the TLRs were processed by removing water molecules by Maestro v-11.3. The web tools dock the protein–ligand complex by performing compact bond docking, clustering of the lowest energy structure, and energy minimization. Based on the center, low energy score, and cluster members, the best docked complex has been chosen [73]. The vaccine complex’s structure was visualized and interaction analyzed by Maestro v-11.3 [74].

### Molecular dynamic simulation

To examine the stability and other dynamics properties of constructed vaccines (V1 and V2) and vaccine-receptor complexes (TLR3-V2, TLR4-V1, and TLR8-V2), 100-ns molecular dynamic simulation [75] was carried out individually using the Schrödinger-Desmond module [76]. The multiepitope vaccine construct was generated through homology modeling and validation; on the other hand, the vaccine receptor was selected through molecular docking. First, each vaccine and vaccine-receptor complex were pre-processed by a protein preparation wizard [77] in Schrodinger-maestro by assigning bond orders, filling the missing side chains, optimizing H-bond, removing water, and minimizing the structure using the OPLS3e force field [78]. For running the MD simulation, a simulation system was created to mimic the original system where the protein structure was positioned. In this regard, a system builder panel was used to build a simulation system. Orthorhombic box shape was used in 30 × 30 × 30 Å^3^ sizes and an OPLS3e force field [78] was applied. Each system was neutralized by adding a different number of Na + and Cl − automatically to this software [79]. Lastly, the SPC water model [80] and 0.15 NaCl were introduced to the simulation box [81]. Finally, using the NPT ensemble, 300.0 k temperature, and 1.01325 bar pressure, a 100-ns MD simulation was run, and the system was relaxed before the simulation began [82]. The simulation trajectory was recorded every 100 ps and approximately the number of frames was 1000. Furthermore, RMSD, RMSF, rGyr, H-bond, and energy values were calculated to evaluate the stability and nature of these complexes. The complete molecular dynamic simulation was run on a Linux (Ubuntu-20.04.1 LTS) computer with an Intel Core i7-10700 K processor, 3200 MHz DDR4 RAM, and an RTX 3080 DDR6 8704 CUDA core GPU.

### Immune simulation for vaccine

The immune simulation-based experiments denote the effective vaccine activity and the level of the vaccine-mediated immune response [83]. Hence, the in silico-based immune simulation was performed with the C-ImmSim v10.1 web server (http://www.cbs.dtu.dk/services/C-ImmSim-10.1/) [84]. For investigating or measuring molecule binding in the context of immune complexes, the server executes immunological simulations based on Miyazawa and Jernigan protein–protein potential measurements [84]. The minimum interval between two doses of vaccines has been previously recommended as 4 weeks. As a result, the computational strategy of administering the vaccine is steps 1, 84, and 168, where one time-step in real life is equivalent to 8 h. In addition, the likely immune response in the pathogen-infected area was assessed using 12 more injections as repeated antigen exposure. Each dose contained 1000 vaccine particles in both situations, and the simulation was run for 1050-time steps (350 days). The Simpson index (D) was used to determine the variety of immunological responses [85–87].

## Results

### Assessment of protein antigenicity

Antigenicity of the protein sequence was predicted, and the highest score found for M glycoprotein (UniProtKB ID: A0A068JCX3) was 0.5496. The S nucleoprotein (UniProtKB ID: A0A193H6Z7) showed a score of 0.3205 and L RNA-dependent RNA polymerase (UniProtKB ID: A0A286MG42) showed a score of 0.4362. The results are tabulated in Table 1.

**Table 1:** Highest antigenic protein selection from the glycoprotein, RdRp, and nucleoprotein of the CCHF virus

NCBI ID	UniProtKB	Protein	Segment	Antigen score
AIE16132.1	A0A068JCX3	Glycoprotein (G)	M	0.5496
ASW20656.1	A0A286MG42	RdRp	L	0.4362
ANN89748.1	A0A193H6Z7	Nucleoprotein (N)	S	0.3205

### Selection of potent immunogenic epitopes

A total of 806 CTL epitopes (9 AA) were predicted through NetCTL 1.2 webserver using 12 supertypes. Among them, 563 epitopes were further selected to assess the immunogenicity, toxicity, allergenicity, and antigenicity score. Based on all these parameters along with an antigenicity threshold value of 0.75, a total of 20 epitopes have been selected (Supplementary Table S1). Following this, MHC-I binding alleles were also predicted for CTL epitopes through the IEDB web server based on the highest number of binding alleles (Supplementary Table S1). Finally, 4 CTL epitopes were selected, with no toxicity and allergenicity, the highest immunogenicity, and antigenicity. Besides, the four epitopes show good binding alleles.

Two thousand ninety-nine epitopes were found for HTL epitopes and 29 of HTL (15 AA) epitopes and their respective MHC-II alleles have been screened out that can induce IFN-γ, IL-4, and IL-10 cytokines (Supplementary Table S2). Finally, based on the highest number of MHC-II binding alleles, four HTL epitope has been selected (Tables 2 and 3).

**Table 2:** Selected CTL epitope properties

Protein name	Super type	CD8 epitope	Combined score	Immunogenicity score	Toxicity	Antigenicity (score)	Allergenicity	Alleles
Glycoprotein	B44	LEMEIILTL	2.0682	0.31195	No	Yes (1.038)	No	9
Glycoprotein	B44	REIEINVLL	2.0797	0.29582	No	Yes (0.526)	No	8
Glycoprotein	B39	YTSICLFVL	1.6693	0.1402	No	Yes (0.615)	No	14
Glycoprotein	B27	KRSSWLVIL	1.5033	0.09769	No	Yes (1.439)	No	6

**Table 3:** Tope selected HTL epitope properties

Protein name	Peptide	Percentile rank	IFN-γ	IFN 4	IFN 10	Antigenicity (score)	Toxicity	Allergenicity	Alleles
Glycoprotein	LFFMFGWRILFCFKC	0.21	+ ve	Inducer	IL10 inducer	Yes (1.301)	Non-toxin	No	18
RDRP	ETVNLIFFAALSAPW	0.44	+ ve	Inducer	IL10 inducer	Yes (0.632)	Non-toxin	No	6
RDRP	SEELYNIRLQHLELS	0.05	+ ve	Inducer	IL10 inducer	Yes (1.502)	Non-toxin	No	10
RDRP	TVNLIFFAALSAPWC	0.44	+ ve	Inducer	IL10 inducer	0.574 (yes)	Non-toxin	No	8

### Analysis of population coverage

**Fig. 1:**
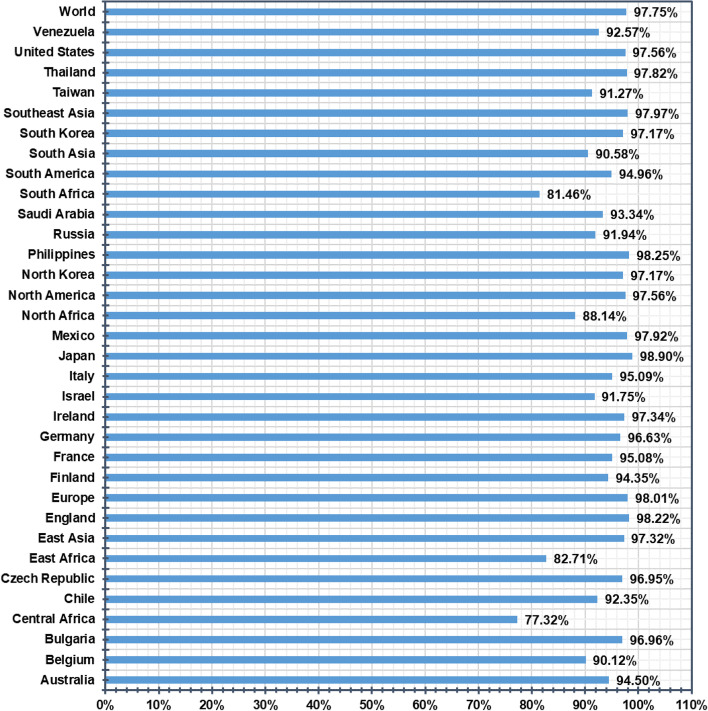
Worldwide population coverage is predicted based on the selected CTL and HTL epitopes

**Fig. 2:**
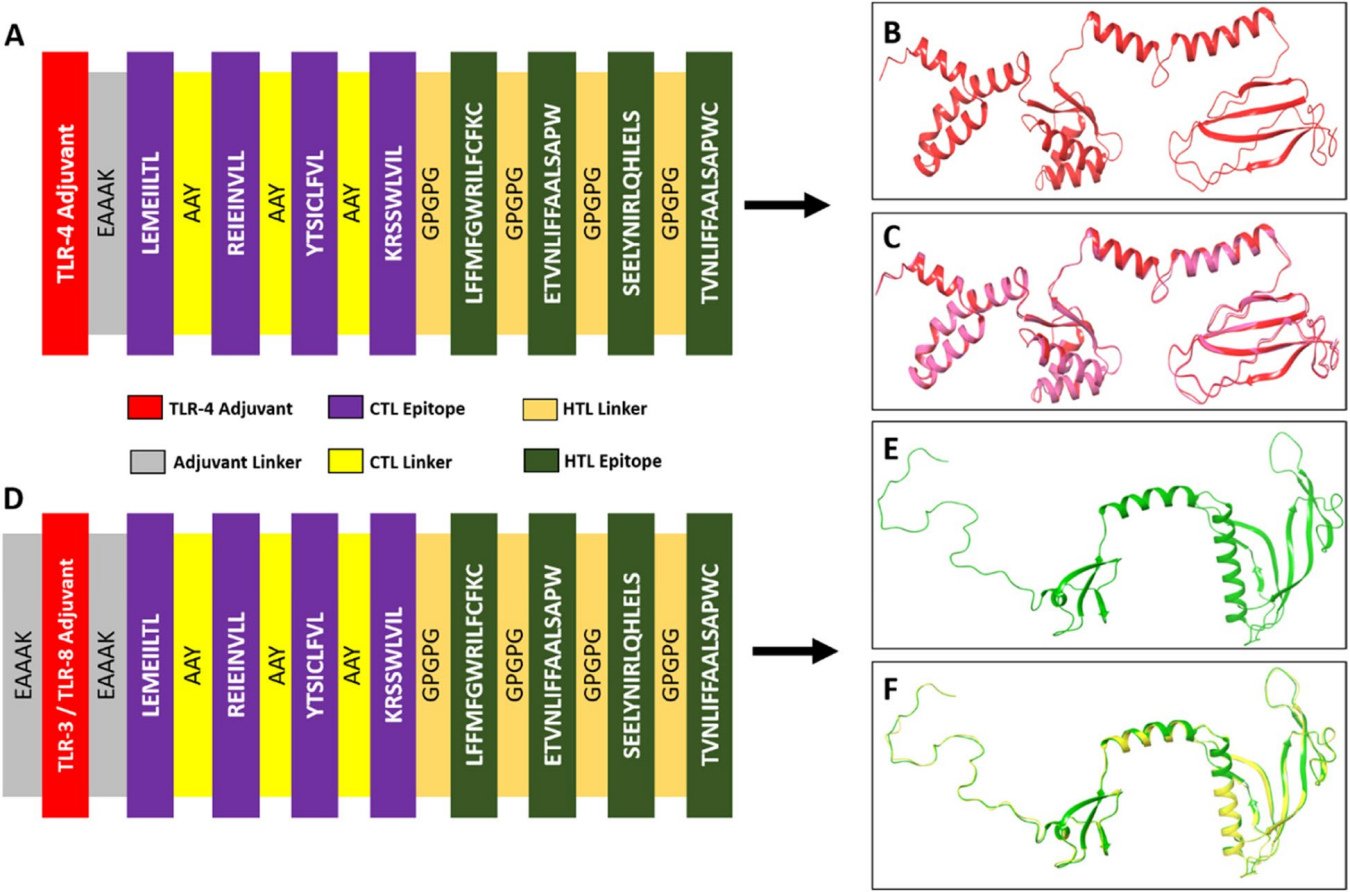
The vaccine construction, modeling, and refinement. **A** TLR4 Adjuvant-vaccine (V1), **B** V1 model, **C** V1 model refined and crude superimposition, **D** TLR3-8 Adjuvant-vaccine (V2), **E** V2 vaccine structure, and **F** V2 refined and initial model superimposition

The population coverage was estimated for the obtained HTL and CTL epitopes. The epitope binding alleles were also considered during the evaluation of the coverage study and are presented in Fig. 2. A high percentage (97.75%) of population coverage worldwide was found for both HTL and CTL epitopes. The interactions of epitopes with a high number of HLA alleles have been found across different countries such as North Africa (88.14%), East Africa (82.71%), USA (97.56%), Spain (97.19%), North America (97.56%), Europe (98.01%), Philippines (98.25%), and Southeast Asia (97.97%). The population coverage study result indicates that the designed vaccine with these epitopes could be a rational vaccine for most of the population in the world (Fig. 1 and Supplementary Table S3).

### Vaccine construct, modeling, and refinement

Each of the 4 CTL and HTL epitopes has been integrated for constructing the vaccine model. Two vaccine fragments with adjuvants have been constructed. One is the TLR4 Adjuvant-vaccine (V1) and another is the TLR3/TLR8 Adjuvant-vaccine (V2) as shown in Figs. 2A and 2D. TLR4 adjuvant -50S ribosomal protein (TLR4 agonist, red) has been combined with EAAAK linker (blue) and linked with CTL epitopes. The AAY (yellow) and GPGPG (orange) linkers have been joined with CTL and HTL epitopes, respectively. The consequences of the vaccine construct are depicted in Fig. 2A. Similarly, TLR3/TLR8 Adjuvant-vaccine (V2) has been also constructed (Fig. 2D). The final construct consists of 260 AA and 199 AA for V1 and V2, respectively.

The tertiary structure has been modeled by performing homology modeling through the RaptorX server. Both 100% amino acid (AA) residues of V1 (260 AA) and V2 (199 AA) have been chosen as input for three and two domains, respectively. Among them, based on the fittest *p*-value, the best template has been chosen. The fittest *p*-value has been chosen as < 10e − 3^70^. The best template for V1 and V2 models has been found as 1dd3A (*p*-value 7.13e − 05) and 4wsbA (*p*-value 6.19e − 05), respectively (Supplementary Table S4). The 3D structure of the initial vaccine model was visualized by the Maestro v-11.3 (Fig. 2B, E). Fifteen percent and 5% positions of AA have been found as disordered. The α-helix, β-sheets, and random coil for the V1 model were found as 38%, 18%, and 43%, respectively. Similarly, the V2 model showed 39% of α-helix, 24% of β-sheets, and 36% of a random coil. The overall homology modeling score has been tabulated in Supplementary Table S4.

The initial V1 and V2 vaccine structures were refined by the Galaxy Refine server, which generated five models for each vaccine. Among V1 and V2 of the refined 3D structures, model 1 was selected for better quality, and the structure was visualized by Maestro v-11.3 (Fig. 2C, F).

### Validation of the vaccine structure

The initial and refined tertiary structures of V1 and V2 were validated by PROCHECK and ProSA-web. Before refinement, the Ramachandran plot of the V1 model showed 92.40% AA residues in most favored regions, 6.30% AA residues in additional allowed regions, 0.40% AA residues in generously allowed regions, and 0.90% AA residues in disallowed regions, while after refinement it showed 94.60% AA residues in most favored regions, 4.00% AA residues in additional allowed regions, null (0%) AA residues in generously allowed regions, and 1.30% AA residues in disallowed regions (Table 4). In contrast, before and after refinement, the Ramachandran plot of the V2 model showed 87.50% and 92.90%AA residues in the most favored regions, respectively. Moreover, the refined V1 and V2 models show *z* scores of − 3.73 and − 2.89, respectively. The overall results of pre-refinement and post-refinement are in Table 4. Since the refined model shows a better score than the crude model, we consider the refined model for further steps.

**Table 4:** Validation of the V1 and V2 vaccine structure through Ramachandran and *z*-score

Vaccine	Residue covered region	Crude	Refine
V1 vaccine	Residues in most favored regions [A, B, L]	92.40%	94.60%
	Residues in additional allowed regions [a, b, l, p]	6.30%	4.00%
	Residues in generously allowed regions [~ a, ~ b, ~ l, ~ p]	0.40%	0.00%
	Residues in disallowed regions	0.90%	1.30%
	z score	− 3.44	− 3.73
V2 vaccine	Residues in most favored regions [A, B, L]	87.50%	92.90%
	Residues in additional allowed regions [a, b, l, p]	9.50%	6.00%
	Residues in generously allowed regions [~ a, ~ b, ~ l, ~ p]	3.00%	0.00%
	Residues in disallowed regions	0.00%	1.20%
	z score	− 2.93	− 2.89

### Physicochemical property evaluation of vaccine construct

Physiochemical attributes of V1 and V2 constructs have been evaluated. V1 and V2 belong to the molecular weight of 27,611.20 and 21,730.75, respectively. The immunogenicity was found for V1 and V2 as 3.87883 and 1.90516, respectively. Our results also show that the instability index for V1 and V2 constructs is respectively 30.24 and 38.12 that denotes they are stable. Other physiochemical entities have been tabulated in Table 5. The formulated vaccine showed positive physicochemical properties, strong immunogenic and antigenic, and negative toxic and allergenic effects on the human body.

**Table 5:** Physicochemical property evaluation of vaccine construct

Evaluating parameters	TLR4 adjuvant-vaccine (V1)	TLR3/TLR8 adjuvant-vaccine (V2)
Number of amino acids	260	199
Molecular weight	27,611.20	21,730.75
Chemical formula	C1273H2001N305O361S8	C999H1567N261O257S12
Theoretical PI	4.75	9.37
Total number of atoms	3948	3096
Total number of negatively charged **residues (Asp + Glu)**	34	13
Total number of positively charged residues (Arg + Lys)	23	25
Extinction coefficient (at 280 nm in **H**_**2**_**O)**	29,700	38,555
Estimated half-life (mammalian reticulocytes**, in vitro)**	30 h	30 h
**Estimated half-life (yeast cells, in vivo)**	> 20 h	> 20 h
**Estimated half-life (*Escherichia coli*, in vivo)**	> 10 h	> 10 h
Instability index	30.24 (stable)	38.12 (stable)
Aliphatic index	106.73	96.28
Grand average of hydropathicity (GRAVY)	0.418	0.292
Immunogenicity	3.87883	1.90516
Antigenicity (VaxiJen)	0.5174 (antigen)	0.6203 (antigen)
**Antigenicity (ANTIGENpro)**	0.209754	0.292126
Allergenicity (AllerTOP)	Non-allergen	Non-allergen
Allergenicity (Allergenfp)	Allergen	Non-allergen
Solubility (SolPro)	0.965681 (soluble)	0.601 (soluble)
Solubility (Protein-Sol)	0.669 (soluble)	0.703733 (soluble)

### Binding affinity of the vaccine-receptor complex

The molecular docking evaluation study showed the binding efficacy of vaccine structures to their corresponding receptor. The different poses of the dock score are shown in Supplementary Table S5, and the docked complex was selected based on the center, low energy score, and cluster members. The TLR-4 complex (TLR4-V1) showed a binding score of − 1001.4 at the center while the lowest energy was found as − 1166.3. The TLR-3 complex (TLR3-V2) showed a binding score of − 1089 at the center while the lowest energy was found as − 1258.7. The TLR-8 complex (TLR8-V2) showed a binding score of − 1106.2 at the center while the lowest energy was found as − 1342.5. The results have been tabulated in Table 6. These vaccine complexes showed good binding affinity and strong interaction.

**Table 6:** Docking score between V1 and TLR4 receptor, V2 and TLR3 receptor, and V2 and TLR8 receptor

Vaccine complex	Representative	Weighted score
TLR-3 complex (TLR3-V2)	Centre	− 1089
	Lowest energy	− 1258.7
TLR-4 complex (TLR4-V1)	Centre	− 1001.4
	Lowest energy	− 1166.3
TLR-**8** c**omplex (TLR8-V2)**	Centre	− 1106.2
	Lowest energy	− 1342.5

### Interaction analysis of V1 and V2 complexes

**Fig. 3:**
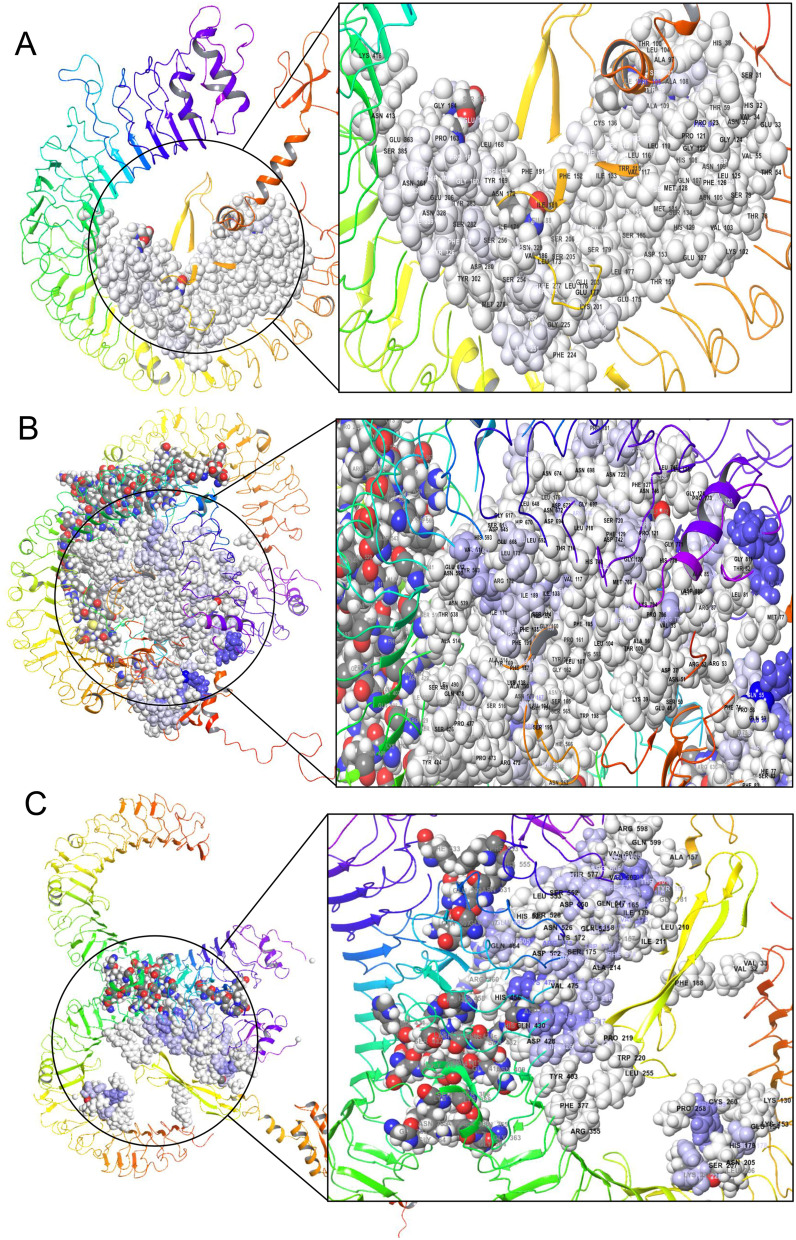
Representing the molecular interaction between vaccine construct and host TLRs receptors that help to activate and trigger innate immune responses resulting in enhancing the potential protective immune response of the host. The complex interaction of the vaccine (V) constructs with the host viral recognition receptors TLRs has been depicted after molecular docking. Herein, showing the interactions between **A** TLR3 and vaccine construct 2 (TLR3-V2), **B** TLR8 and vaccine construct 2 (TLR8-V2), **C** TLR4 and vaccine construct 1 (TLR4-V1). The cartoon structures of proteins represent the TLRs receptors, whereas the ball shape of the protein represents the vaccine constructs

The TLR-4 complex (TLR4-V1) formed 20 hydrogen bonds between receptor and vaccine at Glu485:Arg173, His299:Trp259, His426:Ala218/Pro219, Tyr451:Ser217/Leu216, and Lys477:Leu216/Ser 217/Ala215, etc. The complete results of HB formation are presented in Supplementary Table S6. Additionally, the interaction of TLR3-vaccine complex (TLR3-V2) formed 28 hydrogen bonds at various amino acid sites between receptor and vaccine such as Asp81:Tyr98, Glu110:Arg112, Ser132:114Ser, and His156: Ser113, etc. (S7). Interestingly, 49 HB were found such as Arg541:Pro163/Glu167/Gly164, Tyr567:Glu167, Lys699/Glu88/Tyr86, and Gln55:Lys50, etc., in the case of TLR8-vaccine complex (TLR8-V2) presented in S8. The V2 vaccine interacted with the 92AA and 198AA residues of the TLR3/TLR8 receptor, and this result was visualized (Fig. 3A, B). Additionally, the V1 vaccine interacted with the 68AA residues of the TLR4 human receptor, and this result was illustrated in Fig. 3D. V2 shows the highest AA interaction and hydrogen bond with the TLR8 receptor; however, V2-TLR3 and V1-TLR4 show good interaction and hydrogen bond (Fig. 3 and Table S6-S8).

### Assessment of biophysical properties by MD simulation

To evaluate the vaccine and vaccine-receptor complex’s stability, flexibility, compactness, and energy level, 100-ns simulations were executed. Furthermore, the RMSD of alpha-carbon, RMSF, H-bond, and superimposition was analyzed.

#### RMSD analysis

**Fig. 4:**
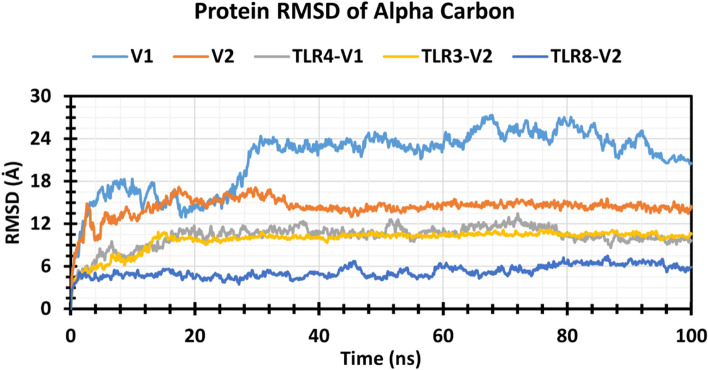
Graph represented the RMSD values of the vaccine and vaccine complex. V1 (light blue), V2 (orange), TLR4-V1 (ash), TLR3-V2 (yellow), and TLR8-V2 (blue) are represented here

The root mean square deviation (RMSD) value is used to compute the mean variation in dislocation of a selection of atoms for a particular frame of protein of protein–protein complex to a reference frame. In the graph, V1 showed more RMSD value and fluctuation compared to the other vaccine V2 (Fig. 4). At 28 ns, the RMSD value of V1 increased to 24 Å from 17 Å, and the rest of the simulation time, its RMSD value remained around 24 Å. On the other hand, V2 shows slight fluctuation till 20 ns, and it was stabilized until the end of the simulation. Among three vaccines (V1, V2)-receptor (TLR3, TLR4, TLR8) complexes, the TLR8-V2 complex has shown the lowest RMSD with an average RMSD value of 5.24 Å and RMSD value confined in the 7.485–1.133 Å range. However, the other two complexes (TLR4-VI, TLR3-V2) have shown an average value of RMSD 10.24 Å and 9.76 Å, respectively. Although TLR4-VI and TLR3-V2 showed greater RMSD compared to the TLR8-V2 complex, they have shown a little amount of fluctuation. From 0 to 16 ns, TLR3-V2 RMSD was increasing but the rest of the simulation time was stable, and TLR4-V1 had almost the same pattern though TLR4-V1 had shown a little bit more fluctuation than TLR3-V2 (Fig. 4).

#### RMSF analysis

**Fig. 5:**
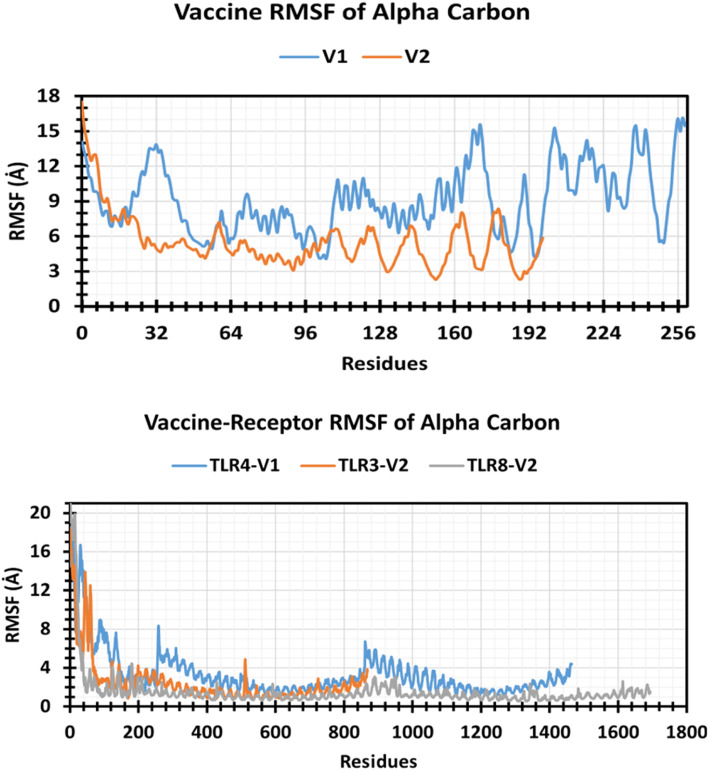
Graphs represented the RMSF values of vaccines and vaccine-receptor complexes, where V1 and V2 represented the light blue and orange colors, respectively. The TLR4-V1, TLR3-V2, and TLR8-V2 represented the light blue, orange, and ash colors, respectively

The root-mean-square fluctuation (RMSF) is calculated to examine the change of structural flexibility of a protein in a specific amino acid residue. In the graph, the V1 and V2 RMSF value was plotted, and it showed that V2 has less RMSF value than V1 (Fig. 5A). In contrast, TLR8-V2 has shown the lowest RMSF value between two other (TLR4-V1, TLR3-V2) complexes, which may indicate the constant binding interaction among receptor-ligand complex (Fig. 5B). Consequently, more fluctuated regions in the graph signify that degree of flexibility increased in the receptor and vaccine complexes (Fig. 5).

#### Hydrogen bond

**Fig. 6:**
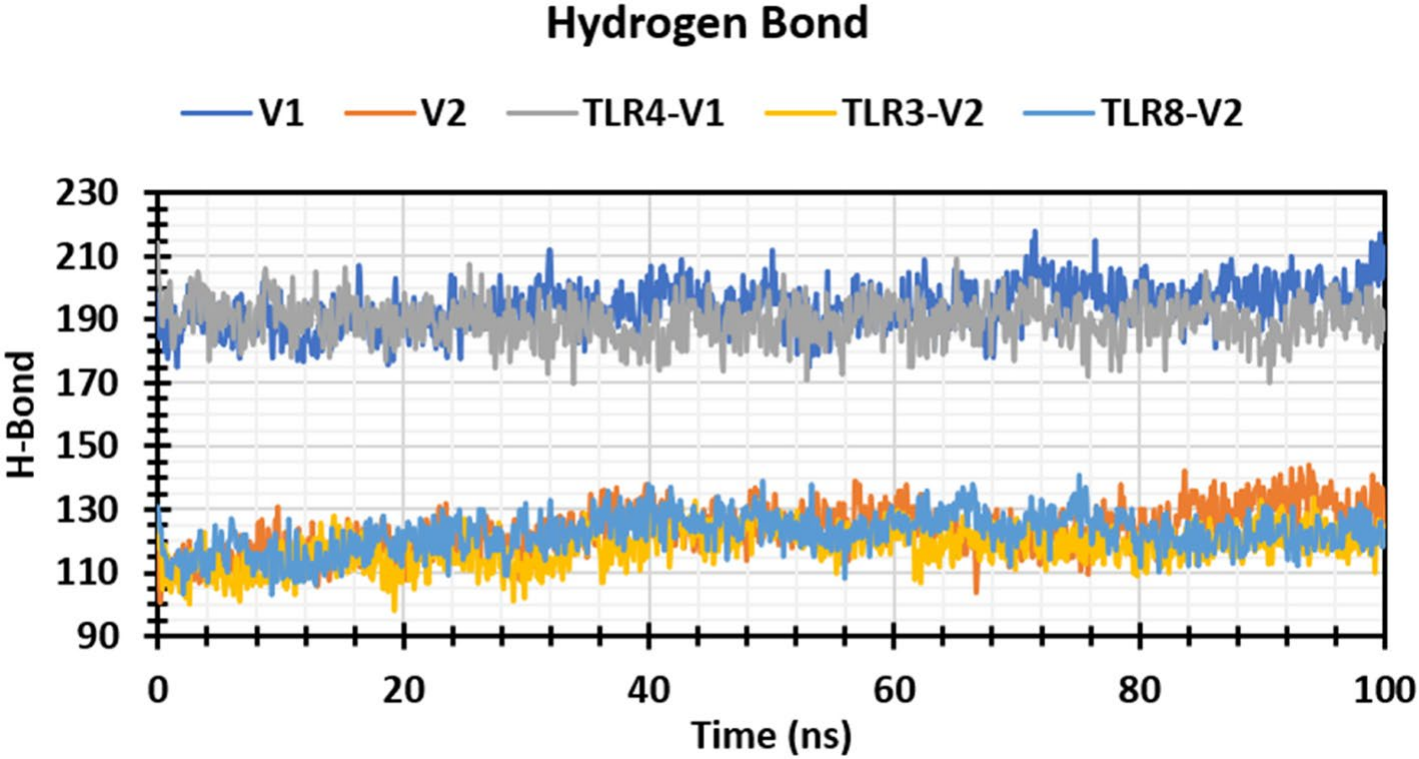
Graph represented the hydrogen bond number of the vaccine and vaccine complex. The V1, V2, TLR4-V1, TLR3-V2, and TLR8-V2 represented the blue, orange, ash, yellow, and light blue colors, respectively

Vaccine candidates binding to the desired receptor binding site rely heavily on hydrogen bonding. The number of hydrogen bonds in a vaccine candidate can help to represent it, which has a big impact on vaccine binding and adsorption. Therefore, the number of hydrogen bonds of the selected vaccine V1, V2, and receptor-vaccine complex structure including TLR4-V1, TLR3-V2, and TLR8-V2 computed for systems by investigating configurations of 100 ns represented in Fig. 6. During the 100-ns simulation run, hydrogen bond numbers were computed from the start to the finish timings to monitor each hydrogen bond. Until the simulation time of 100 ns, all the vaccine structures and vaccine-receptor complex structures established multiple hydrogen bonds ranging from 90 to 210. As a result, all the complex structures will significantly improve the strength and stability of the vaccine–receptor contact.

#### Superimposition of vaccine complexes through MD simulation

**Fig. 7:**
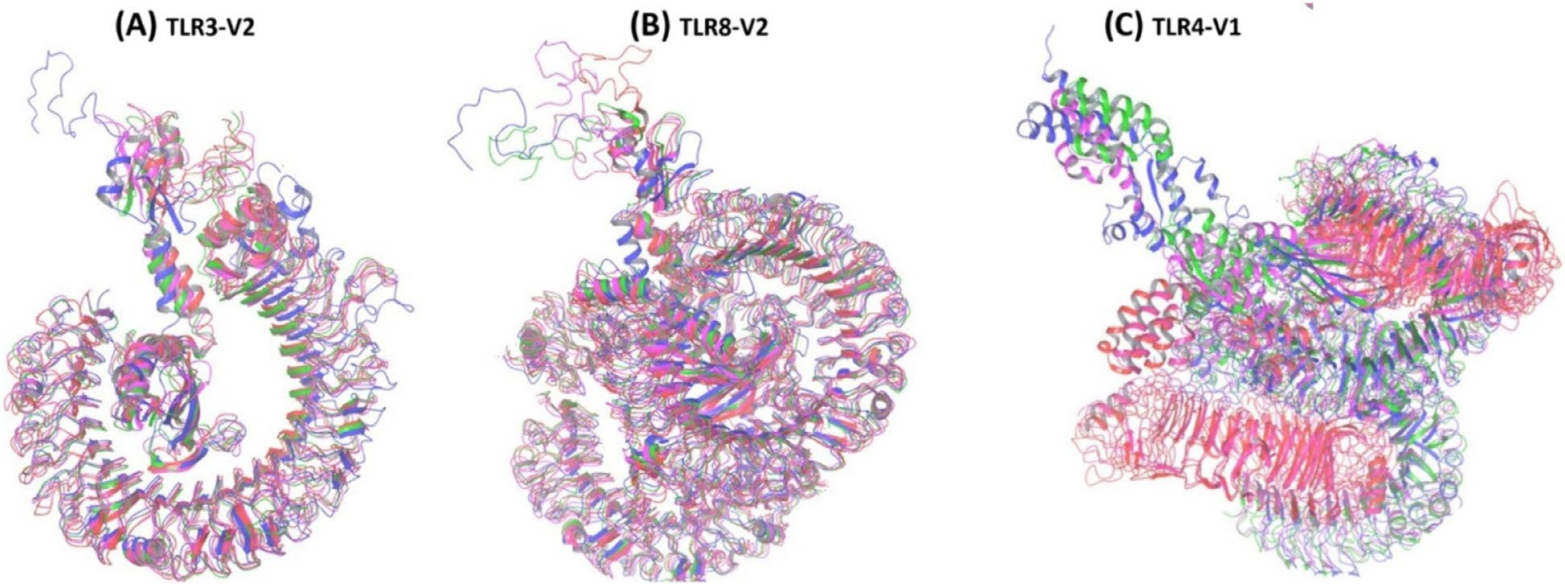
Superimposition of vaccine complexes through MD simulation. **A** TLR3-V2, **B** TLR8-V2, and **C** TLR4-V1

Protein 3D superposition for 0, 25, 50, 75, and 100 ns was applied, and superimpose protein 3D structures were applied to identify similarities of protein folds. The coordinate of a mobile protein is transformed (superposed) so that the backbone lies over the backbone of a reference protein. The superimpose structure of the protein has shown low flexibility shown in Fig. 7.

### Evaluation of in silico immune simulation response

**Fig. 8:**
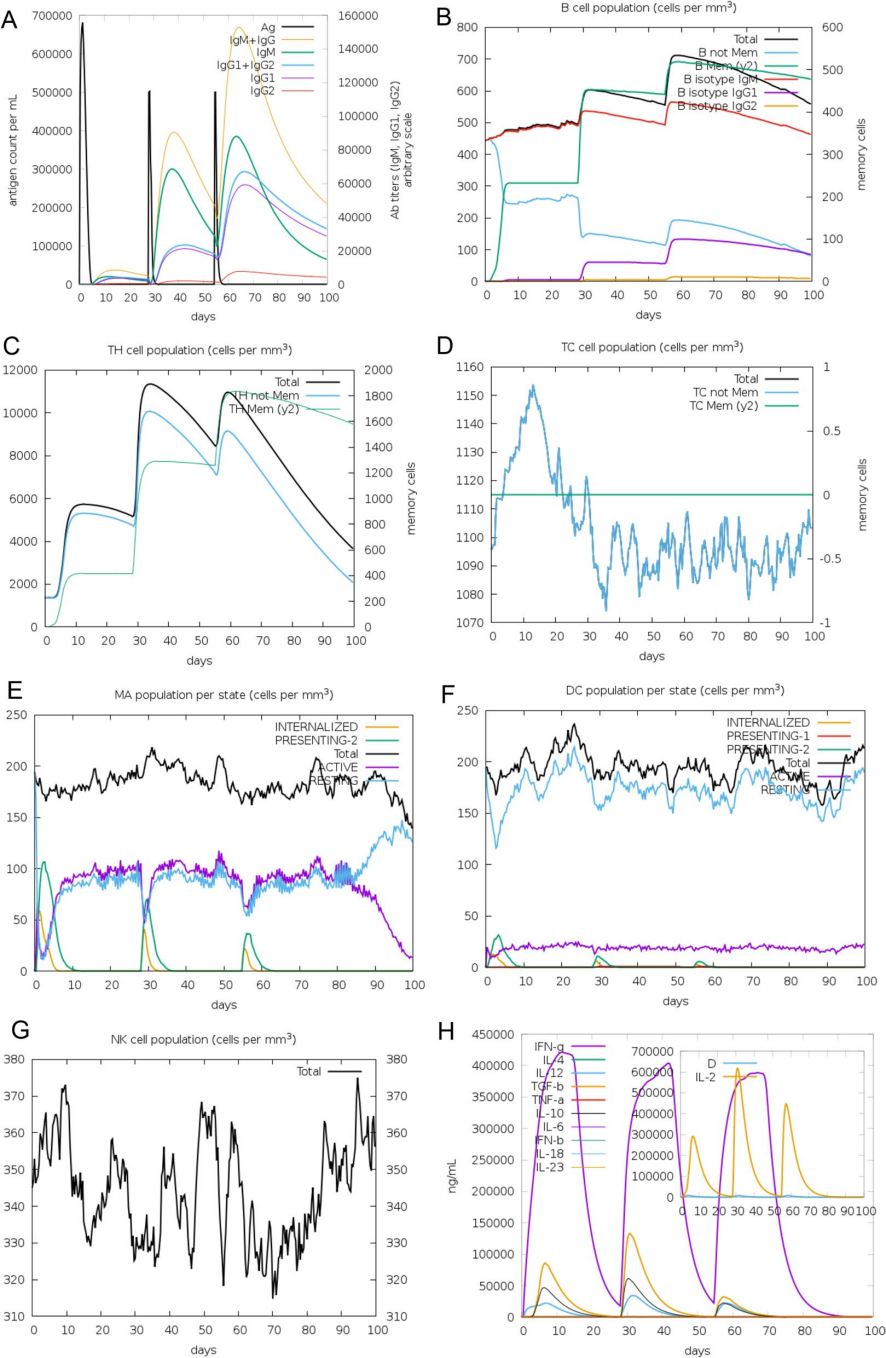
Immune simulation causing V1 antigen. The graph shows the condition of different kinds of immune cells. **A** Immune response causing vaccine antigen invading; **B** memory B cell formation; **C** helper T cell formation; **D** cytotoxic T cell population; **E**–**G** macrophage, dendritic, and natural killer cell population per state; and **H** concentration of cytokines and interleukins

The in silico immune response evaluation generated in C-ImmSim immune simulator is presented in Fig. 8. That showed that after internalization of the initial antigen for TLR3/8, it induced all the primary immune responses like IgM, IgG, and IgG1 + IgG2 production (~ 8–29th days). After 30 days, the Ag level was reduced, and the antibody production becomes increased, which was found to last for ~ 55 days. After that, IgM, IgG, and IgG1 + IgG2 production increased significantly with a consistent level of Ag. Throughout the 100 days, the response IgG1 level was found to not have significant changes (Fig. 8A). In addition, there were several total functional B-cell formations with memory B cell formation, and B cell isotype IgM/IgG1 presentation was also significantly increased (Fig. 8B). Similarly, the parallel immune response like the higher response of Th (helper) and Tc (cytotoxic) cell populations with corresponding T cell memory development was also observed (Fig. 8C, D). Additionally, post-exposure, the total macrophage population was increased, whereas DC activity was consistent (Fig. 8E, F). NK cell activity fluctuated throughout the duration (Fig. 8G). Higher levels of IFNγ and IL-2 and moderate levels of TGFβ, IL-10, and IL-18 were also evident. In addition, a lower Simpson index (*D*) indicates greater diversity (Fig. 8H).

**Fig. 9:**
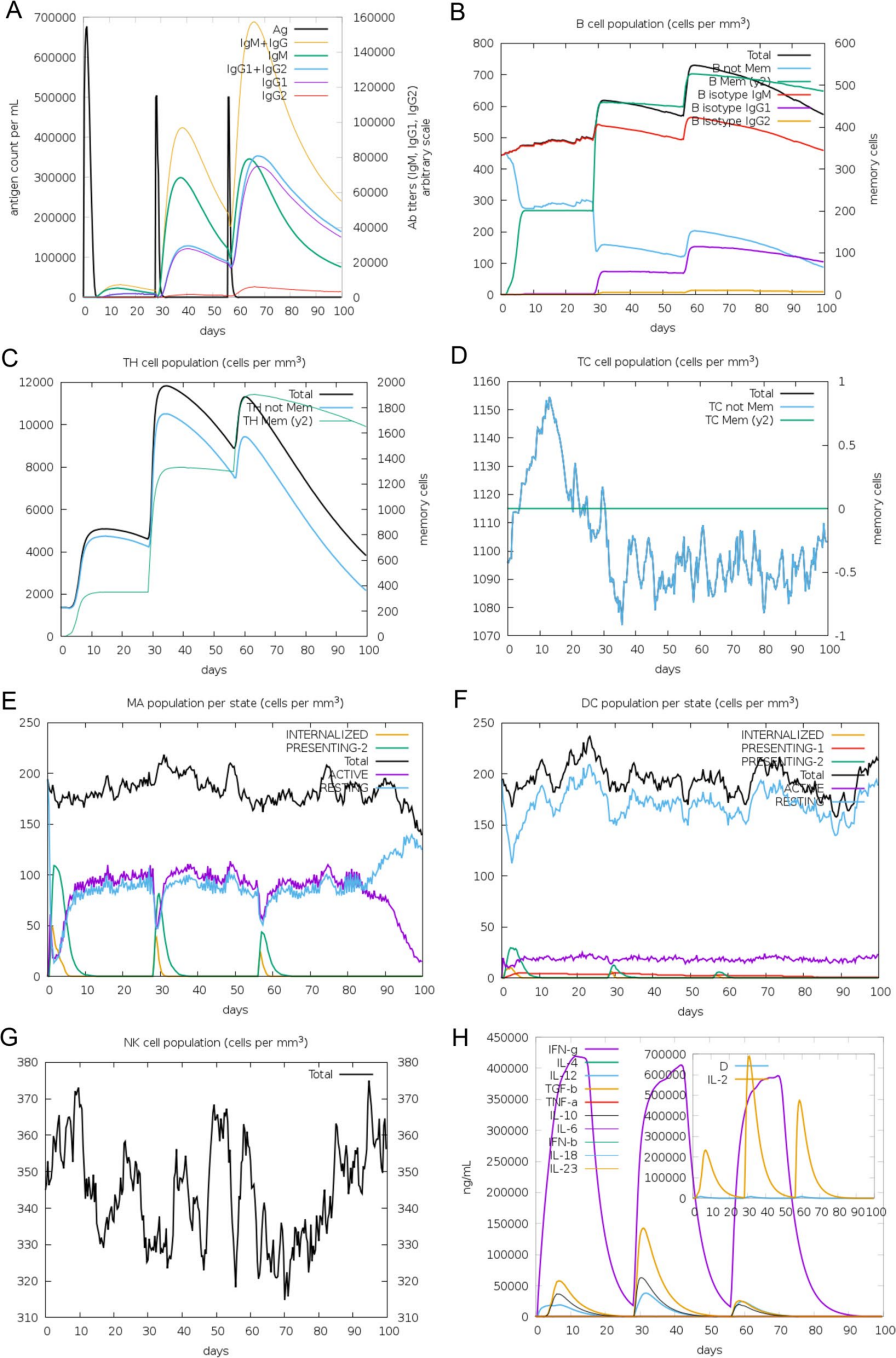
Immune simulation causing V1 antigen. The graph shows the condition of different kinds of immune cells. **A** Immune response causing vaccine antigen invading; **B** memory B cell formation; **C** helper T cell formation; **D** cytotoxic T cell population; **E**–**G** macrophage, dendritic, and natural killer cell population per state; and **H** concentration of cytokines and interleukins

Similarly, after the initial administration of antigen for TLR4, it induced all the primary immune responses like IgM, IgG, and IgG1 + IgG2 production (~ 8–29th days). After 30 days, the Ag level was reduced, and the antibody production becomes increased, which was found to last for ~ 55 days. After that, IgM, IgG, and IgG1 + IgG2 production also increased significantly with a consistent level of antigen (Ag). Unlikely TLR3/8 epitopes and TLR4 epitopes manifested non-significant IgG2 levels (Fig. 9A). Likely TLR3/8 epitopes and TLR4 epitopes also showed the increased level of several total functional B-cell formations with memory B cell formation; B cell isotypes IgM/IgG1 and MHC-II presentation were also significantly increased (Fig. 9B). Simultaneously, Th (helper) and Tc (cytotoxic) cell populations with corresponding T cell memory development were also increased (Fig. 9C, D) and total macrophage population has a consistent DC activity (Fig. 9E, F), and fluctuated NK cell activity was observed throughout the duration (Fig. 9G). Higher levels of IFN-γ and IL-2 and moderate levels of TGFβ, IL-10, and IL-12 were also evident. In addition, a lower Simpson index (*D*) indicates greater diversity (Fig. 9H).

## Discussion

The World Health Organization (WHO) addressed CCHFV as a hemorrhagic fever outbreak-causing virus that has a fatality rate of up to 40%. But, to date, no specific therapeutics have been developed against the virus, and developing a novel treatment option is an urgent issue. At this instant, multiepitope-based vaccines that are being developed by different computational methods can be used in the treatment of viral diseases. The computational methods for designing multiepitope-based vaccine have many advantages. The techniques can screen out the whole genome to find the potent immunogenic epitope with a maximum immunogenic response. Also, the epitopes identified through the methods do not elicit adverse viral pathogenicity [17]. Therefore, the study aimed to identify different potential epitopes from the CCHFV protein for constructing multiepitope-based vaccine candidates to treat the viral disease.

To identify potential epitopes of the virus, the three negative sense RNA proteins of the virus namely N protein, glycoprotein (G) precursor, and RdRp were retrieved to evaluate the antigenic properties through different computational tools. Determination of antigenic region is important because it can evoke both humoral and cellular immunogenicity [88]. The computational evaluation identified that the G protein and RdRp were more antigenic than the N protein. Previously, an *in vivo* study found more immunogenic properties of the N protein in the virus [89]. But our sequence-based antigenic prediction suggested (Table 1) G and RdRp are more antigenic than N protein. The length of the AA sequence of RdRp and G protein is longer than that of the N protein. Therefore, it could be due to the presence of more antigenic motifs in RdRp and G protein than in N protein. However, Dowall et al. found that the N protein fails to confer protection against lethal viral disease [90]. So, the study utilizes the two (G and RdRp) proteins for identifying potential epitopes against the virus. We identified the best (3 to 4) HTL and CTL epitopes for each protein to formulate the vaccine candidates with appropriate linkers. The specific and appropriate linkers were selected in this study to maintain the cleavability, flexibility, and rigidity of the vaccine candidates. The linker of the vaccine is an essential part of the vaccine required to preserve the immunogenicity of the vaccine and help it to work independently [91, 92]. It can also help to amplify the vaccine stability and optimize the pattern of expression [93]. Using an appropriate length of linkers as well as Ag density can be important for APC function, DC cell uptake, and the response of CD8 + T cells [94]. Hence, the study utilizes a different length of the linker as well as an adjuvant to optimize the functional activity of the vaccine construct [95]. The attached adjuvant not only helps in evoking humoral and cellular immunity but also maintains the longevity of vaccines [96]. Ultimately, the V1 and V2 vaccines were found to be 260 and 199 long amino acids. Moreover, another pivotal physical property of the candidate vaccine is solubility. Therefore, the designed vaccine was considered for predicting the solubility property by utilizing a solubility assessing tool for determining the solubility quality to become soluble inside the *E. coli* host [97]. Our predicted candidate vaccine passed this parameter. The candidate vaccine was also evaluated for PI, and we found it was acidic. Similarly, we evaluated the instability index of the vaccine by availing server tools that showed the stable nature of the protein. On the other hand, the GRAVY value and aliphatic index represent the hydrophobic and thermostable nature of the vaccine, consecutively [98, 99]. Hence, all the physiochemical properties and scores are endowed with the feasibility of the CCCHFV vaccine. The vaccine’s effectiveness depends on the population in which the vaccination is used [86], and our designed vaccine covers the world population very well, with an overall coverage of 97.75%. The 3D structure was predicted that is followed in terms of the lowest binding energy score; the models were taken for refining. The z score of the validated 3D structure was found acceptable [100]. Ramachandran’s plot also showed the acceptable and disallowed regions of the vaccine construct [87]. TLRs are the essential mediators of the inflammatory pathway that help initiate signaling cascades and activate the innate immune system [101]. Mainly four TLRs (TLR-3,4,7, and 8) broadly act as viral antigenic receptors and play a crucial role against viral infections [102] whereas TLR-1,2,5,6,9–12 primarily work against bacterial infection [102]. TLR3, which senses viral dsRNA and structured RNA, is associated with acute CCHF, and enhances the activity of the protein, can stimulate innate immune responses [103–106]. Therefore, the activity of these receptors with the desired vaccine candidate has been observed in this study. The activity of TLR-3,4, and 8 and the vaccine construct was evaluated based on molecular docking simulation. The structure of the vaccine and receptor were retrieved to gain more knowledge about the binding interactions and strength of the complex structure [107]. As the vaccine construct had both CTL and HTL epitopes, it might cause the host’s corresponding immune cells to become activated, which could then cause other immune cells to become activated through complex signaling [108]. It was determined from the immune simulation study that our proposed vaccine candidate might provide a suitable immune response in subsequent exposure following the primary injection. Additionally, these vaccinations can improve B and T cell memory. Furthermore, persistent IL-2 and IFN production was observed. The vaccine candidate underwent a molecular dynamics simulation investigation to confirm their stability under atomistic settings. The simulation results obtained by combining the RMSD and RMSF descriptors from trajectory data are correlated with the structural rigidity of the vaccination complexes. For the majority of the simulation duration, the RMSD and RMSF profile of the vaccine candidate vaccine-receptor complexes was satisfactory. According to these findings, the vaccination complexes are more stable and less mobile under the simulated settings.

## Conclusions

In recent years, CCHFV outbreaks have occurred in various countries. It has been an emerging topic in infectious medical science due to its high fatality rate, lack of promising treatments, and lack of effective vaccination. In the current study, using computational techniques, potential T- and B-cell epitopes were identified and examined for their greater conservancy and immunological features, both antigenic and immunogenic, and non-toxic. The designed multiepitope vaccines are projected to have immune-dominating attributes and also high population coverage. Additionally, our constructed vaccines were able to potentially bind with immune receptors TLR3/8 and TLR4 and exert an immunogenic response against CCHF. Based on these results, we may conclude that our vaccine construct may provide a new therapeutic window for treating CCHF infection. However, further *in vitro* and *in vivo* experiments are required to validate our computationally formulated vaccine as an effective vaccine against CCHF.

## Supplementary Information

**Additional file 1:** **Table S1.** Screening of CTLepitopes by Immunoginecity, Toxicity, Allergenicity, Antigenicity score, andAlleles. **Table S2.** Screening of HTLepitopes by IFN-ƴ, IL-4 and IL-10 cytokines, antigenicity score, and Alleles. **Table S3.** Population coverage of CTLand HTL epitopes. **Table S4:** Maintaining criterias of vaccine modelling.Table S5: Molecular docking of vaccine-receptor. **Table S6.** Interaction of TLR4-Vaccine Complex (TLR4-V1). Table S7. Interaction ofTLR3-Vaccine Complex (TLR3-V2). **Table S8.** Interaction of TLR8-VaccineComplex (TLR8-V2).

## Data Availability

In the article and supplementary file.
