# Supplementary material for: In silico formulation of a next-generation multiepitope vaccine for use as a prophylactic candidate against Crimean-Congo hemorrhagic fever
Source: BMC Med. 2023 Feb 1;21:36. doi: 10.1186/s12916-023-02750-9 (PMC9891764; doi:10.1186/s12916-023-02750-9)
Supplement: Supplementary file 1 — Additional file 1: Table S1. Screening of CTLepitopes by Immunoginecity, Toxicity, Allergenicity, Antigenicity score, andAlleles. Table S2. Screening of HTLepitopes by IFN-ƴ, IL-4 and IL-10 cytokines, antigenicity score, and Alleles. Table S3. Population coverage of CTLand HTL epitopes. Table S4: Maintaining criterias of vaccine modelling.Table S5: Molecular docking of vaccine-receptor. Table S6. Interaction of TLR4-Vaccine Complex (TLR4-V1). Table S7. Interaction ofTLR3-Vaccine Complex (TLR3-V2). Table S8. Interaction of TLR8-VaccineComplex (TLR8-V2). [file 12916_2023_2750_MOESM1_ESM.docx]

**Table S1. Screening of CTL epitopes by Immunogenicity, Toxicity, Allergenicity, Antigenicity score, and Alleles.**

| Name | Supertype | Epitope | Combined Score | Immunogenicity | Toxicity | Antigenicity | Allergenicity | | Allele |
| --- | --- | --- | --- | --- | --- | --- | --- | --- | --- |
|  |  |  |  |  |  |  | AllergenFP | AllerTop |  |
| glycoprotein | B39 | HREIEINVL | 2.4858 | 0.40786 | NO | 0.75 | NO | NO | 4 |
| glycoprotein | B44 | LEMEIILTL | 2.0682 | 0.31195 | NO | 1.038 | NO | NO | 9 |
| glycoprotein | B44 | REIEINVLL | 2.0797 | 0.29582 | NO | 0.526 | NO | NO | 8 |
| glycoprotein | B39 | YTSICLFVL | 1.6693 | 0.1402 | NO | 0.615 | NO | NO | 14 |
| glycoprotein | B27 | KRSSWLVIL | 1.5033 | 0.09769 | NO | 1.439 | NO | NO | 6 |
| glycoprotein | B7 | APISHGETV | 1.4077 | 0.04731 | NO | 0.88 | NO | NO | 3 |
| glycoprotein | B62 | LSRGSEVVL | 0.7566 | 0.04301 | NO | 0.589 | NO | NO | 2 |
| nucleoprotein | A3 | ASALVEATK | 0.899 | 0.19169 | NO | 0.641 | NO | NO | 2 |
| RDRP | B39 | FNHREIADL | 0.9348 | 0.33134 | NO | 1.731 | NO | NO | 2 |
| RDRP | A1 | VTDIVVGAI | 1.4576 | 0.27164 | NO | 1.063 | NO | NO | 2 |
| RDRP | B39 | YHSIAELTM | 2.0951 | 0.22589 | NO | 0.61 | NO | NO | 3 |
| RDRP | B39 | RNLDWTQVI | 0.7701 | 0.19732 | NO | 2.111 | NO | NO | 3 |
| RDRP | B27 | SQLLIEIKR | 0.9031 | 0.19541 | NO | 0.918 | NO | NO | 3 |
| RDRP | A3 | KLLELLGIK | 1.3713 | 0.18227 | NO | 0.841 | NO | NO | 4 |
| RDRP | B44 | PESVEAVPV | 0.8195 | 0.15053 | NO | 0.966 | NO | NO | 1 |
| RDRP | B39 | DHLDVGGLL | 0.7566 | 0.11294 | NO | 0.923 | NO | NO | 3 |
| RDRP | A3 | HLDVGGLLR | 0.9685 | 0.0978 | NO | 0.741 | NO | NO | 4 |
| RDRP | A24 | DYEDLALAL | 0.9468 | 0.09435 | NO | 1.73 | NO | NO | 4 |
| RDRP | A3 | TLILRPESK | 1.0861 | 0.05984 | NO | 0.844 | NO | NO | 2 |
| RDRP | B7 | RPTGLTLPT | 1.4164 | 0.0566 | NO | 0.506 | NO | NO | 3 |

*RNA-dependent RNA polymerase = RDRP

**Table S2. Screening of HTL epitopes by IFN-ƴ, IL-4 and IL-10 cytokines, antigenicity score, and alleles.**

| Name | Peptide | percentile_rank | IFN-gamma | IFN_4prediction | IFN_10prediction | Antigenecity | Toxicity | Allergenecity | |
| --- | --- | --- | --- | --- | --- | --- | --- | --- | --- |
|  |  |  |  |  |  |  |  | AlrgenFP | AltTOP |
| glycoprotein | AMPKTSLCFYIVERE | 2.2 | +ve | Inducer | Inducer | 0.8872 | No | No | No |
| RDRP | DWLRDGKRRGNLELA | 1.2 | +ve | Inducer | Inducer | 0.5273 | No | No | No |
| RDRP | ETVNLIFFAALSAPW | 0.44 | +ve | Inducer | Inducer | 0.6319 | No | No | No |
| RDRP | FLNWRVNMDIRASDC | 2.1 | +ve | Inducer | Inducer | 1.0395 | No | No | No |
| glycoprotein | GFLSMDSGYFSAKCY | 1.3 | +ve | Inducer | Inducer | 1.1907 | No | No | No |
| RDRP | GNFLNRSSRDHFISI | 1.4 | +ve | Inducer | Inducer | 0.9043 | No | No | No |
| RDRP | GNSYDFIWTTQMIAN | 1.8 | +ve | Inducer | Inducer | 0.5702 | No | No | No |
| RDRP | ISTQKVRSYLKAGTA | 0.4 | +ve | Inducer | Inducer | 0.6084 | No | No | No |
| glycoprotein | KAFSAMPKTSLCFYI | 0.34 | +ve | Inducer | Inducer | 0.5604 | No | No | No |
| glycoprotein | KTSLCFYIVEREYCK | 2.2 | +ve | Inducer | Inducer | 0.9426 | No | No | No |
| glycoprotein | LFFMFGWRILFCFKC | 0.21 | +ve | Inducer | Inducer | 1.3099 | No | No | No |
| RDRP | MLRFGMLAGLSRLVC | 0.66 | +ve | Inducer | Inducer | 0.6865 | No | No | No |
| glycoprotein | MPKTSLCFYIVEREY | 2.2 | +ve | Inducer | Inducer | 0.9346 | No | No | No |
| RDRP | NPRFNISDYFEIVRQ | 0.27 | +ve | Inducer | Inducer | 0.603 | No | No | No |
| glycoprotein | NSTSLETSLSIEAPW | 2.5 | +ve | Inducer | Inducer | 0.7097 | No | No | No |
| glycoprotein | PKTSLCFYIVEREYC | 2.1 | +ve | Inducer | Inducer | 0.9767 | No | No | No |
| RDRP | QLQMLRFGMLAGLSR | 0.66 | +ve | Inducer | Inducer | 0.8725 | No | No | No |
| RDRP | QMLRFGMLAGLSRLV | 0.66 | +ve | Inducer | Inducer | 0.5389 | No | No | No |
| RDRP | RDHFISIVSGLNLVY | 1.2 | +ve | Inducer | Inducer | 1.0552 | No | No | No |
| RDRP | SEELYNIRLQHLELS | 0.05 | +ve | Inducer | Inducer | 1.5024 | No | No | No |
| RDRP | SFLNWRVNMDIRASD | 2.1 | +ve | Inducer | Inducer | 1.3883 | No | No | No |
| RDRP | SGNSYDFIWTTQMIA | 2.5 | +ve | Inducer | Inducer | 0.6776 | No | No | No |
| RDRP | SYDFIWTTQMIANSN | 1.7 | +ve | Inducer | Inducer | 0.8493 | No | No | No |
| RDRP | TVNLIFFAALSAPWC | 0.44 | +ve | Inducer | Inducer | 0.5737 | No | No | No |
| RDRP | VKGSLKKRLKFMNIH | 0.71 | +ve | Inducer | Inducer | 1.2248 | No | No | No |
| RDRP | VTNPRFNISDYFEIV | 0.27 | +ve | Inducer | Inducer | 0.9109 | No | No | No |
| RDRP | YDFIWTTQMIANSNF | 2.3 | +ve | Inducer | Inducer | 0.9416 | No | No | No |
| RDRP | YIQVLQQYRCLEVIN | 0.73 | +ve | Inducer | Inducer | 0.7321 | No | No | No |
| RDRP | YVTNPRFNISDYFEI | 0.27 | +ve | Inducer | Inducer | 0.6418 | No | No | No |

*RNA-dependent RNA polymerase = RDRP

**Table S3. Population coverage of CTL and HTL epitopes**

| Area | Class combined (Coverage) |
| --- | --- |
| World | 97.75% |
| Austria | 94.43% |
| Belgium | 90.12% |
| Bulgaria | 96.96% |
| Chile | 92.35% |
| Croatia | 93.39% |
| Czech Republic | 96.95% |
| East Asia | 97.32% |
| England | 98.22% |
| Europe | 98.01% |
| Finland | 94.35% |
| France | 95.08% |
| Germany | 96.63% |
| Ireland | 97.34% |
| Israel | 91.75% |
| Italy | 95.09% |
| Japan | 98.90% |
| Mexico | 97.92% |
| Morocco | 94.48% |
| North America | 97.56% |
| North Korea | 97.17% |
| Philippines | 98.25% |
| Poland | 96.56% |
| Portugal | 92.45% |
| Russia | 91.94% |
| Russia Siberian | 91.31% |
| Saudi Arabia | 93.34% |
| South America | 94.96% |
| South Asia | 90.58% |
| South Korea | 97.17% |
| Southeast Asia | 97.97% |
| Spain | 97.19% |
| Sudan | 90.18% |
| Sweden | 94.16% |
| Taiwan | 91.27% |
| Thailand | 97.82% |
| United States | 97.56% |
| Venezuela | 92.57% |
| Australia | 94.50% |
| Central Africa | 77.32% |
| East Africa | 82.71% |
| North Africa | 88.14% |
| South Africa | 81.46% |
| West Africa | 79.51% |

**Table S4:** Maintaining criterias of vaccine modelling

| **Properties** | **V1 Vaccine** | **V2 Vaccine** |
| --- | --- | --- |
| Best Template | 1dd3 | 4wsb |
| p-value | 7.13E-05 | 6.19E-05 |
| Residues Coverage | 100% | 100% |
| Alpha-Helix | 38% | 39% |
| Beta-Sheet | 18% | 24% |
| Random Coil | 43% | 36% |

**Table S5**: Molecular docking of vaccine-receptor.

| **Cluster** | **Representative** | **TLR3-V2** | **TLR4-V1** | **TLR8-V2** |
| --- | --- | --- | --- | --- |
| 0 | Center | -1089 | -1001.4 | -1208.5 |
| 0 | Lowest Energy | -1258.7 | -1166.3 | -1413.3 |
| 1 | Center | -1058.3 | -1060.8 | -1204.6 |
| 1 | Lowest Energy | -1296.2 | -1132.5 | -1484.4 |
| 2 | Center | -1089.9 | -1028.7 | -1315.3 |
| 2 | Lowest Energy | -1251.8 | -1115.4 | -1506 |
| 3 | Center | -1005.7 | -1020.3 | -1145 |
| 3 | Lowest Energy | -1216.6 | -1154.5 | -1314.9 |
| 4 | Center | -978.3 | -1053.2 | -1093.6 |
| 4 | Lowest Energy | -1018.8 | -1166.8 | -1233.5 |
| 5 | Center | -968 | -1119 | -1222.4 |
| 5 | Lowest Energy | -1129.1 | -1157.3 | -1429.2 |
| 6 | Center | -948.4 | -1141.1 | -1045.4 |
| 6 | Lowest Energy | -1056.5 | -1141.1 | -1483.8 |
| 7 | Center | -941.8 | -1093.3 | -1122.8 |
| 7 | Lowest Energy | -1078.1 | -1159.2 | -1225 |
| 8 | Center | -984.7 | -1003.5 | -1096 |
| 8 | Lowest Energy | -1082.4 | -1176.8 | -1184.9 |
| 9 | Center | -957.7 | -1052.6 | -1364.1 |
| 9 | Lowest Energy | -1022.7 | -1125.2 | -1364.1 |
| 10 | Center | -1057.1 | -1020.2 | -1541.2 |
| 10 | Lowest Energy | -1057.1 | -1124.7 | -1541.2 |
| 11 | Center | -1037.2 | -1006.4 | -1107.5 |
| 11 | Lowest Energy | -1037.2 | -1168.2 | -1171.3 |
| 12 | Center | -1094.5 | -970.5 | -1186.7 |
| 12 | Lowest Energy | -1246.4 | -1138.3 | -1186.7 |
| 13 | Center | -1062.3 | -1125.1 | -1022.7 |
| 13 | Lowest Energy | -1062.3 | -1125.1 | -1150.6 |
| 14 | Center | -1052.8 | -1102.5 | -1082.4 |
| 14 | Lowest Energy | -1178.3 | -1140.4 | -1148.5 |
| 15 | Center | -1012.7 | -976.5 | -1029.4 |
| 15 | Lowest Energy | -1054 | -1103.8 | -1248.5 |
| 16 | Center | -941.1 | -975 | -1300.3 |
| 16 | Lowest Energy | -1021 | -1046 | -1531.6 |
| 17 | Center | -999.3 | -998.2 | -1077.2 |
| 17 | Lowest Energy | -1098.6 | -1086.3 | -1204.5 |
| 18 | Center | -993.8 | -1066.6 | -1076.4 |
| 18 | Lowest Energy | -993.8 | -1122.1 | -1076.4 |
| 19 | Center | -941.4 | -986.3 | -1136.7 |
| 19 | Lowest Energy | -1037.5 | -1094.6 | -1145.4 |
| 20 | Center | -1079.2 | -1014.6 | -1133.8 |
| 20 | Lowest Energy | -1079.2 | -1150.7 | -1277.2 |
| 21 | Center | -1003.3 | -1181.8 | -1035.2 |
| 21 | Lowest Energy | -1044.4 | -1181.8 | -1110 |
| 22 | Center | -1043 | -1061.2 | -1118.2 |
| 22 | Lowest Energy | -1069.6 | -1109.6 | -1228.4 |
| 23 | Center | -963.2 | -1015.9 | -1037.3 |
| 23 | Lowest Energy | -1076.4 | -1064.4 | -1179.7 |
| 24 | Center | -962.3 | -971.2 | -1042.5 |
| 24 | Lowest Energy | -1142.9 | -1151.1 | -1115.6 |
| 25 | Center | -1144.4 | -981.7 | -1269.8 |
| 25 | Lowest Energy | -1144.4 | -1229.1 | -1515.5 |
| 26 | Center | -979.7 | -983.1 | -1043.9 |
| 26 | Lowest Energy | -1142.6 | -1185.8 | -1506.3 |
| 27 | Center | -941 | -1002.4 | -1145.9 |
| 27 | Lowest Energy | -1091.4 | -1101.9 | -1145.9 |
| 28 | Center | -976.9 | -1007.3 | -1045.3 |
| 28 | Lowest Energy | -1029.1 | -1126.8 | -1292.6 |
| 29 | Center | -1028.9 | -1033.7 | -1233.4 |
| 29 | Lowest Energy | -1141.8 | -1042.6 | -1233.4 |

**Table S6.** Interaction of TLR4-Vaccine Complex (TLR4-V1)

| **Receptor** | **Vaccine** | **Specific Interactions** | **# HB** |
| --- | --- | --- | --- |
| A:485:Glu | C:173:Arg | 2x hb to C:173:Arg | 2 |
| B:154:Glu | C:260:Cys |  | 0 |
| B:178:Glu | C:259:Trp | 1x clash to C:259:Trp | 0 |
| B:205:Asn | C:259:Trp |  | 0 |
| B:207:Ser | C:259:Trp |  | 0 |
| B:229:His | C:259:Trp | 1x hb to C:259:Trp | 1 |
| B:377:Phe | C:220:Trp |  | 0 |
| B:403:Tyr | C:218:Ala |  | 0 |
| B:426:His | C:218:Ala, C:219:Pro | 1x hb to C:218:Ala | 1 |
| B:428:Asp | C:217:Ser |  | 0 |
| B:451:Tyr | C:217:Ser, C:216:Leu | 1x clash to C:216:Leu 1x hb to C:217:Ser | 1 |
| B:477:Lys | C:216:Leu, C:217:Ser, C:215:Ala | 1x hb, 1x clash to C:215:Ala 1x clash to C:217:Ser | 1 |
| B:500:Phe | C:214:Ala,C:176:Trp,C:215:Ala | 1x clash to C:176:Trp | 0 |
| B:505:Gln | C:174:Ser | 1x hb to C:174:Ser | 1 |
| B:523:Gln |  |  | 0 |
| B:524:Val | C:213:Phe, C:176:Trp | 1x clash to C:176:Trp | 0 |
| B:547:Gln | C:213:Phe, C:211:Ile |  | 0 |
| B:548:Val | C:213:Phe | 1x clash to C:213:Phe | 0 |
| B:550:Asp | C:177:Leu |  | 0 |
| B:552:Ser | C:177:Leu |  | 0 |
| B:553:Leu | C:173:Arg, C:172:Lys |  | 0 |
| B:573:Phe | C:180:Leu, C:179:Ile | 1x clash to C:180:Leu | 0 |
| B:575:Asn | C:177:Leu | 1x clash to C:177:Leu | 0 |
| B:577:Thr | C:177:Leu |  | 0 |
| B:578:Gln | C:173:Arg, C:168:Leu | 1x hb to C:173:Arg | 1 |
| B:598:Arg | C:180:Leu |  | 0 |
| B:599:Gln | C:180:Leu |  | 0 |
| B:601:Leu | C:180:Leu | 1x clash to C:180:Leu | 0 |
| B:602:Val | C:178:Val, C:160:Tyr, C:180:Leu |  | 0 |
| B:603:Glu | C:160:TyrC:178:ValC:177:LeuC:164:Cys | 1x hb to C:160:Tyr1x clash to C:164:Cys1x clash to C:177:Leu2x clash to C:178:Val | 1 |
| B:605:Glu | C:160:Tyr, C:161:Thr, C:164:Cys | 1x clash to C:161:Thr | 0 |
| B:606:Arg | C:164:Cys, C:177:Leu, C:168:Leu | 1x hb to C:164:Cys | 1 |
| C:160:Tyr | B:603:Glu, B:605:Glu, B:602:Val | 1x hb to B:603:Glu | 1 |
| C:161:Thr | B:605:Glu | 1x clash to B:605:Glu | 0 |
| C:164:Cys | B:606:Arg, B:603:Glu, B:605:Glu | 1x clash to B:603:Glu 1x hb to B:606:Arg | 1 |
| C:168:Leu | B:578:Gln, B:606:Arg |  | 0 |
| C:172:Lys | B:553:Leu |  | 0 |
| C:173:Arg | A:485:Glu, B:578:Gln, B:553:Leu | 2x hb to A:485:Glu 1x hb to B:578:Gln | 3 |
| C:174:Ser | B:505:Gln | 1x hb to B:505:Gln | 1 |
| C:176:Trp | B:500:Phe, B:524:Val | 1x clash to B:500:Phe 1x clash to B:524:Val | 0 |
| C:177:Leu | B:603:Glu, B:606:Arg, B:575:Asn, B:550:Asp,B:577:Thr, B:552:Ser | 1x clash to B:575:Asn 1x clash to B:603:Glu | 0 |
| C:178:Val | B:603:Glu, B:602:Val | 2x clash to B:603:Glu | 0 |
| C:179:Ile | B:573:Phe |  | 0 |
| C:180:Leu | B:573:Phe,B:601:Leu, B:599:Gln, B:598:Arg, B:602:Val | 1x clash to B:573:Phe 1x clash to B:601:Leu | 0 |
| C:211:Ile | B:547:Gln |  | 0 |
| C:213:Phe | B:547:Gln, B:524:Val, B:548:Val | 1x clash to B:548:Val | 0 |
| C:214:Ala | B:500:Phe |  | 0 |
| C:215:Ala | B:477:LysB:500:Phe | 1x hb, 1x clash to B:477:Lys | 1 |
| C:216:Leu | B:477:Lys, B:451:Tyr | 1x clash to B:451:Tyr | 0 |
| C:217:Ser | B:451:Tyr, B:477:Lys,B:428:Asp | 1x hb to B:451:Tyr 1x clash to B:477:Lys | 1 |
| C:218:Ala | B:426:His, B:403:Tyr | 1x hb to B:426:His | 1 |
| C:219:Pro | B:426:His |  | 0 |
| C:220:Trp | B:377:Phe |  | 0 |
| C:259:Trp | B:178:Glu,B:207:Ser, B:205:Asn, B:229:His | 1x clash to B:178:Glu 1x hb to B:229:His | 1 |
| C:260:Cys | B:154:Glu |  | 0 |

**Table S7.** Interaction of TLR3-Vaccine Complex (TLR3-V2)

| **Receptor** | **Vaccine** | **Specific Interactions** | **# HB** |
| --- | --- | --- | --- |
| A:32:His | B:124:Gly |  | 0 |
| A:34:Val | B:124:Gly, B:122:Gly |  | 0 |
| A:39:His | B:104:Leu, B:101:Ser |  | 0 |
| A:55:Val | B:126:Phe |  | 0 |
| A:57:Asn | B:98:Tyr |  | 0 |
| A:60:Hie | B:101:Ser, B:105:Phe, B:104:Leu | 2x clash to B:101:Ser 1x clash to B:105:Phe | 0 |
| A:79:Ser | B:126:Phe |  | 0 |
| A:81:Asp | B:98:Tyr, B:119:Leu | 1x hb to B:98:Tyr | 1 |
| A:84:Phe | B:105:Phe, B:116:Leu, B:108:Ala | 28x clash to B:105:Phe | 0 |
| A:103:Val | B:126:Phe, B:128:Met |  | 0 |
| A:105:Asn | B:119:Leu, B:117:Val |  | 0 |
| A:107:Gln | B:116:Leu, B:117:Val |  | 0 |
| A:108:His | B:112:Arg, B:108:Ala, B:109:Ala, B:105:Phe, B:114:Ser |  | 0 |
| A:109:Asn | B:112:Arg |  | 0 |
| A:110:Glu | B:112:Arg | 1x hb, 1x salt bridge to B:112:Arg | 1 |
| A:127:Glu | B:128:Met |  | 0 |
| A:129:His | B:117:Val |  | 0 |
| A:131:Met | B:117:Val |  | 0 |
| A:132:Ser | B:114:Ser, B:115:Trp | 1x hb to B:114:Ser | 1 |
| A:153:Asp | B:115:Trp, B:117:Val |  | 0 |
| A:155:Ser | B:115:Trp |  | 0 |
| A:156:His | B:113:Ser, B:115:Trp, B:114:Ser, B:135:Phe | 1x hb to B:113:Ser | 1 |
| A:177:Leu | B:115:Trp |  | 0 |
| A:179:Ser | B:115:Trp |  | 0 |
| A:180:Asn | B:135:Phe, B:115:Trp | 1x clash to B:135:Phe | 0 |
| A:200:Lys | B:174:Gln | 1x hb to B:174:Gln | 1 |
| A:201:Lys | B:133:Ile, B:173:Leu |  | 0 |
| A:203:Glu | B:152:Phe |  | 0 |
| A:227:Phe | B:173:Leu |  | 0 |
| A:229:Asn | B:191:Phe, B:171:Ile |  | 0 |
| A:251:Arg | B:174:Gln, B:175:Hip | 1x hb to B:174:Gln 1x hb to B:175:Hip | 2 |
| A:252:Asn | B:172:Arg, B:173:Leu, B:174:Gln | 1x hb to B:172:Arg | 1 |
| A:254:Ser | B:171:Ile |  | 0 |
| A:278:Met | B:172:Arg |  | 0 |
| A:280:Asp | B:171:Ile |  | 0 |
| A:282:Ser | B:169:Tyr |  | 0 |
| A:283:Tyr | B:169:Tyr |  | 0 |
| A:302:Tyr | B:172:Arg, B:186:Val |  | 0 |
| A:304:Phe | B:170:Asn, B:172:Arg, B:171:Ile | 1x clash to B:172:Arg | 0 |
| A:306:Glu | B:170:Asn, B:169:Tyr |  | 0 |
| A:307:Tyr | B:167:Glu, B:169:Tyr | 1x hb to B:167:Glu | 1 |
| A:326:Tyr | B:159:Trp, B:172:Arg | 1x hb to B:159:Trp | 1 |
| A:328:Asn | B:159:Trp |  | 0 |
| A:330:Lys | B:167:Glu, B:163:Pro B:164:Gly | 2x hb, 1x salt bridge to B:167:Glu | 2 |
| A:359:Hie | B:160:Gly | 1x hb to B:160:Gly | 1 |
| A:361:Asn | B:163:Pro, B:159:Trp |  | 0 |
| A:363:Glu | B:163:Pro, B:164:Gly |  | 0 |
| A:383:Tyr | B:161:Pro, B:163:Pro | 1x hb to B:161:Pro | 1 |
| A:385:Ser | B:163:Pro |  | 0 |
| B:98:Tyr | A:81:Asp, A:57:Asn | 1x hb to A:81:Asp | 1 |
| B:101:Ser | A:60:Hie, A:39:His | 2x clash to A:60:Hie | 0 |
| B:104:Leu | A:39:His, A:60:Hie |  | 0 |
| B:105:Phe | A:84:Phe, A:60:Hie A:108:His | 1x clash to A:60:Hie 28x clash to A:84:Phe | 0 |
| B:108:Ala | A:108:His, A:84:Phe |  | 0 |
| B:109:Ala | A:108:His |  | 0 |
| B:112:Arg | A:110:Glu, A:108:His A:109:Asn | 1x hb, 1x salt bridge to A:110:Glu | 1 |
| B:113:Ser | A:156:His | 1x hb to A:156:His | 1 |
| B:114:Ser | A:132:Ser, A:156:His, A:108:His | 1x hb to A:132:Ser | 1 |
| B:115:Trp | A:179:Ser, A:156:His, A:155:Ser, A:153:Asp, A:177:Leu, A:132:Ser, A:180:Asn |  | 0 |
| B:116:Leu | A:84:Phe, A:107:Gln |  | 0 |
| B:117:Val | A:131:Met, A:105:Asn, A:153:Asp, A:129:His, A:107:Gln |  | 0 |
| B:119:Leu | A:105:Asn, A:81:Asp |  | 0 |
| B:122:Gly | A:34:Val |  | 0 |
| B:124:Gly | A:32:His, A:34:Val |  | 0 |
| B:126:Phe | A:79:Ser, A:55:Val, A:103:Val |  | 0 |
| B:128:Met | A:103:Val, A:127:Glu |  | 0 |
| B:133:Ile | A:201:Lys |  | 0 |
| B:135:Phe | A:180:Asn, A:156:His | 1x clash to A:180:Asn | 0 |
| B:152:Phe | A:203:Glu |  | 0 |
| B:159:Trp | A:326:Tyr, A:328:Asn, A:361:Asn | 1x hb to A:326:Tyr | 1 |
| B:160:Gly | A:359:Hie | 1x hb to A:359:Hie | 1 |
| B:161:Pro | A:383:Tyr | 1x hb to A:383:Tyr | 1 |
| B:163:Pro | A:363:Glu, A:383:Tyr, A:330:Lys, A:361:Asn, A:385:Ser |  | 0 |
| B:164:Gly | A:363:Glu, A:330:Lys |  | 0 |
| B:167:Glu | A:330:Lys, A:307:Tyr | 1x hb to A:307:Tyr 2x hb, 1x salt bridge to A:330:Lys | 3 |
| B:169:Tyr | A:283:Tyr, A:307:Tyr, A:282:Ser, A:306:Glu |  | 0 |
| B:170:Asn | A:304:Phe, A:306:Glu |  | 0 |
| B:171:Ile | A:280:Asp, A:254:Ser, A:229:Asn, A:304:Phe |  | 0 |
| B:172:Arg | A:252:Asn, A:302:Tyr, A:278:Met, A:304:Phe A:326:Tyr | 1x hb to A:252:Asn 1x clash to A:304:Phe | 1 |
| B:173:Leu | A:252:Asn, A:227:Phe, A:201:Lys |  | 0 |
| B:174:Gln | A:200:Lys, A:251:Arg, A:252:Asn | 1x hb to A:200:Lys 1x hb to A:251:Arg | 2 |
| B:175:Hip | A:251:Arg | 1x hb to A:251:Arg | 1 |
| B:186:Val | A:302:Tyr |  | 0 |
| B:191:Phe | A:229:Asn |  | 0 |

**Table S8.** Interaction of TLR8-Vaccine Complex (TLR8-V2)

| **Receptor** | **Vaccine** | **Specific Interactions** | **# HB** |
| --- | --- | --- | --- |
| A:539:Asn | C:159:Trp C:163:Pro | 1x clash to C:159:Trp | 0 |
| A:541:Arg | C:163:Pro C:167:Glu C:164:Gly | 1x hb to C:163:Pro 2x hb, 1x salt bridge to C:167:Glu | 3 |
| A:563:Tyr | C:160:Gly C:163:Pro |  | 0 |
| A:565:Ser | C:164:Gly C:162:Gly C:163:Pro C:165:Ser |  | 0 |
| A:567:Tyr | C:167:Glu C:164:Gly | 1x hb to C:167:Glu | 1 |
| A:593:His | C:161:Pro |  | 0 |
| A:652:Lys | C:88:Glu | 1x salt bridge to C:88:Glu | 0 |
| A:675:Met | C:88:Glu |  | 0 |
| A:699:Lys | C:88:Glu C:86:Tyr C:87:Arg | 1x hb to C:86:Tyr 1x hb, 1x clash to C:88:Glu | 2 |
| A:723:Arg | C:86:Tyr C:85:Ala | 1x clash to C:86:Tyr | 0 |
| B:39:Lys | C:104:Leu |  | 0 |
| B:48:Glu | C:100:Thr |  | 0 |
| B:51:Asn | C:100:Thr C:96:Ala C:97:Ala |  | 0 |
| B:55:Gln | C:50:Lys C:77:Met | 1x hb to C:50:Lys | 1 |
| B:56:Glu | C:41:Arg C:67:Arg C:50:Lys | 2x hb, 1x salt bridge to C:41:Arg 1x hb, 1x salt bridge, 3x clash to C:67:Arg | 3 |
| B:57:Val | C:41:Arg | 1x hb to C:41:Arg | 1 |
| B:59:Gln | C:41:Arg |  | 0 |
| B:76:Thr | C:50:Lys | 1x hb to C:50:Lys | 1 |
| B:77:Hie | C:49:Pro |  | 0 |
| B:79:Thr | C:48:Leu |  | 0 |
| B:80:Asn | C:44:Val C:62:Arg |  | 0 |
| B:81:Glu | C:47:Cyx C:44:Val C:43:Ala C:46:Ser C:45:Leu C:48:Leu C:42:Cys | 1x clash to C:43:Ala 1x clash to C:44:Val 1x hb to C:46:Ser 1x hb to C:47:Cyx | 2 |
| B:84:Gln | C:42:Cys |  | 0 |
| B:123:Asn | C:62:Arg | 1x hb to C:62:Arg | 1 |
| B:262:Asn | C:167:Glu |  | 0 |
| B:472:Arg | C:169:Tyr C:193:Ala |  | 0 |
| B:476:Lys | C:148:Asn C:137:Phe C:150:Ile | 1x hb to C:148:Asn 1x clash to C:150:Ile | 1 |
| B:490:Leu | C:169:Tyr |  | 0 |
| B:539:Asn | C:171:Ile C:172:Arg |  | 0 |
| B:563:Tyr | C:189:Ile C:172:Arg C:152:Phe C:171:Ile |  | 0 |
| B:593:His | C:173:Leu C:189:Ile |  | 0 |
| B:612:Glu | C:115:Trp |  | 0 |
| B:614:Val | C:115:Trp |  | 0 |
| B:643:Arg | C:113:Ser C:115:Trp | 1x clash to C:113:Ser 1x clash to C:115:Trp | 0 |
| B:645:Asp | C:115:Trp |  | 0 |
| B:648:Leu | C:133:Ile |  | 0 |
| B:650:Arg | C:174:Gln C:176:Leu | 1x hb to C:174:Gln | 1 |
| B:668:Glu | C:112:Arg |  | 0 |
| B:672:Asn | C:117:Val |  | 0 |
| B:673:Asp | C:117:Val C:119:Leu |  | 0 |
| B:691:Glu | C:112:Arg | 1x hb to C:112:Arg | 1 |
| B:692:Leu | C:112:Arg |  | 0 |
| B:696:Arg | C:115:Trp C:116:Leu | 1x hb to C:115:Trp 1x clash to C:116:Leu | 1 |
| B:697:Gly | C:128:Met |  | 0 |
| B:699:Lys | C:128:Met | 1x clash to C:128:Met | 0 |
| B:718:Leu | C:105:Phe |  | 0 |
| B:721:Hie | C:119:Leu C:98:Tyr C:120:Gly C:126:Phe C:121:Pro | 1x clash to C:119:Leu | 0 |
| B:722:Asn | C:126:Phe |  | 0 |
| B:723:Arg | C:126:Phe | 1x hb to C:126:Phe | 1 |
| B:740:His | C:105:Phe |  | 0 |
| B:744:Ser | C:98:Tyr | 1x hb to C:98:Tyr | 1 |
| B:745:Ser | C:98:Tyr C:126:Phe C:121:Pro | 1x hb to C:98:Tyr | 1 |
| B:747:Leu | C:126:Phe |  | 0 |
| B:768:Glu | C:101:Ser | 1x hb to C:101:Ser | 1 |
| B:770:His | C:98:Tyr C:101:Ser |  | 0 |
| B:796:Pro | C:104:Leu |  | 0 |
| B:797:Arg | C:97:Ala C:101:Ser C:100:Thr | 1x clash to C:97:Ala 1x hb to C:101:Ser | 1 |
| B:810:Arg | C:78:Glu | 1x salt bridge, 4x clash to C:78:Glu | 0 |
| C:41:Arg | B:57:Val B:56:Glu B:59:Gln | 2x hb, 1x salt bridge to B:56:Glu 1x hb to B:57:Val | 3 |
| C:43:Ala | B:81:Glu | 1x clash to B:81:Glu | 0 |
| C:44:Val | B:81:Glu B:80:Asn | 1x clash to B:81:Glu | 0 |
| C:46:Ser | B:81:Glu | 1x hb to B:81:Glu | 1 |
| C:47:Cyx | B:81:Glu | 1x hb to B:81:Glu | 1 |
| C:50:Lys | B:55:Gln B:76:Thr B:56:Glu | 1x hb to B:55:Gln 1x hb to B:76:Thr | 2 |
| C:62:Arg | B:123:Asn B:80:Asn | 1x hb to B:123:Asn | 1 |
| C:67:Arg | B:56:Glu | 1x hb, 1x salt bridge, 3x clash to B:56:Glu | 1 |
| C:77:Met | B:55:Gln |  | 0 |
| C:78:Glu | B:810:Arg | 1x salt bridge, 4x clash to B:810:Arg | 0 |
| C:85:Ala | A:723:Arg |  | 0 |
| C:86:Tyr | A:699:Lys A:723:Arg | 1x hb to A:699:Lys 1x clash to A:723:Arg | 1 |
| C:87:Arg | A:699:Lys |  | 0 |
| C:88:Glu | A:652:Lys A:699:Lys A:675:Met | 1x salt bridge to A:652:Lys 1x hb, 1x clash to A:699:Lys | 1 |
| C:96:Ala | B:51:Asn |  | 0 |
| C:97:Ala | B:797:Arg B:51:Asn | 1x clash to B:797:Arg | 0 |
| C:98:Tyr | B:744:Ser B:745:Ser B:770:His B:721:Hie | 1x hb to B:744:Ser 1x hb to B:745:Ser | 2 |
| C:100:Thr | B:51:Asn B:48:Glu B:797:Arg |  | 0 |
| C:101:Ser | B:768:Glu B:797:Arg B:770:His | 1x hb to B:768:Glu 1x hb to B:797:Arg | 2 |
| C:104:Leu | B:796:Pro B:39:Lys |  | 0 |
| C:105:Phe | B:740:His B:718:Leu |  | 0 |
| C:112:Arg | B:691:Glu B:692:Leu B:668:Glu | 1x hb to B:691:Glu | 1 |
| C:113:Ser | B:643:Arg | 1x clash to B:643:Arg | 0 |
| C:115:Trp | B:696:Arg B:645:Asp B:643:Arg B:612:Glu B:614:Val | 1x clash to B:643:Arg 1x hb to B:696:Arg | 1 |
| C:116:Leu | B:696:Arg | 1x clash to B:696:Arg | 0 |
| C:117:Val | B:673:Asp B:672:Asn |  | 0 |
| C:119:Leu | B:721:Hie B:673:Asp | 1x clash to B:721:Hie | 0 |
| C:120:Gly | B:721:Hie |  | 0 |
| C:121:Pro | B:745:Ser B:721:Hie |  | 0 |
| C:126:Phe | B:723:Arg B:722:Asn B:745:Ser B:721:Hie B:747:Leu | 1x hb to B:723:Arg | 1 |
| C:128:Met | B:697:Gly B:699:Lys | 1x clash to B:699:Lys | 0 |
| C:133:Ile | B:648:Leu |  | 0 |
| C:137:Phe | B:476:Lys |  | 0 |
| C:148:Asn | B:476:Lys | 1x hb to B:476:Lys | 1 |
| C:150:Ile | B:476:Lys | 1x clash to B:476:Lys | 0 |
| C:152:Phe | B:563:Tyr |  | 0 |
| C:159:Trp | A:539:Asn | 1x clash to A:539:Asn | 0 |
| C:160:Gly | A:563:Tyr |  | 0 |
| C:161:Pro | A:593:His |  | 0 |
| C:162:Gly | A:565:Ser |  | 0 |
| C:163:Pro | A:541:Arg A:563:Tyr A:565:Ser A:539:Asn | 1x hb to A:541:Arg | 1 |
| C:164:Gly | A:565:Ser A:541:Arg A:567:Tyr |  | 0 |
| C:165:Ser | A:565:Ser |  | 0 |
| C:167:Glu | A:567:Tyr A:541:Arg B:262:Asn | 2x hb, 1x salt bridge to A:541:Arg 1x hb to A:567:Tyr | 3 |
| C:169:Tyr | B:472:Arg B:490:Leu |  | 0 |
| C:171:Ile | B:539:Asn B:563:Tyr |  | 0 |
| C:172:Arg | B:563:Tyr B:539:Asn |  | 0 |
| C:173:Leu | B:593:His |  | 0 |
| C:174:Gln | B:650:Arg | 1x hb to B:650:Arg | 1 |
| C:176:Leu | B:650:Arg |  | 0 |
| C:189:Ile | B:563:Tyr B:593:His |  | 0 |
| C:193:Ala | B:472:Arg |  | 0 |
